# Nothing lasts forever: understanding microbial biodegradation of polyfluorinated compounds and perfluorinated alkyl substances

**DOI:** 10.1111/1751-7915.13928

**Published:** 2021-09-27

**Authors:** Lawrence P. Wackett

**Affiliations:** ^1^ Department of Biochemistry, Molecular Biology and Biophysics University of Minnesota St. Paul MN 55108 USA

## Abstract

Poly‐ and perfluorinated chemicals, including perfluorinated alkyl substances (PFAS), are pervasive in today’s society, with a negative impact on human and ecosystem health continually emerging. These chemicals are now subject to strict government regulations, leading to costly environmental remediation efforts. Commercial polyfluorinated compounds have been called ‘forever chemicals’ due to their strong resistance to biological and chemical degradation. Environmental cleanup by bioremediation is not considered practical currently. Implementation of bioremediation will require uncovering and understanding the rare microbial successes in degrading these compounds. This review discusses the underlying reasons why microbial degradation of heavily fluorinated compounds is rare. Fluorinated and chlorinated compounds are very different with respect to chemistry and microbial physiology. Moreover, the end product of biodegradation, fluoride, is much more toxic than chloride. It is imperative to understand these limitations, and elucidate physiological mechanisms of defluorination, in order to better discover, study, and engineer bacteria that can efficiently degrade polyfluorinated compounds.

## Graphical abstract

This review discusses recent knowledge pertaining to the microbial degradation of polyfluorinated compounds and PFAS. It further explores underlying reasons why this biodegradation is so rare in nature and posits prospects for future discovery and implementation.

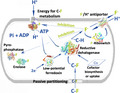

## Introduction

**Fig. 1:**
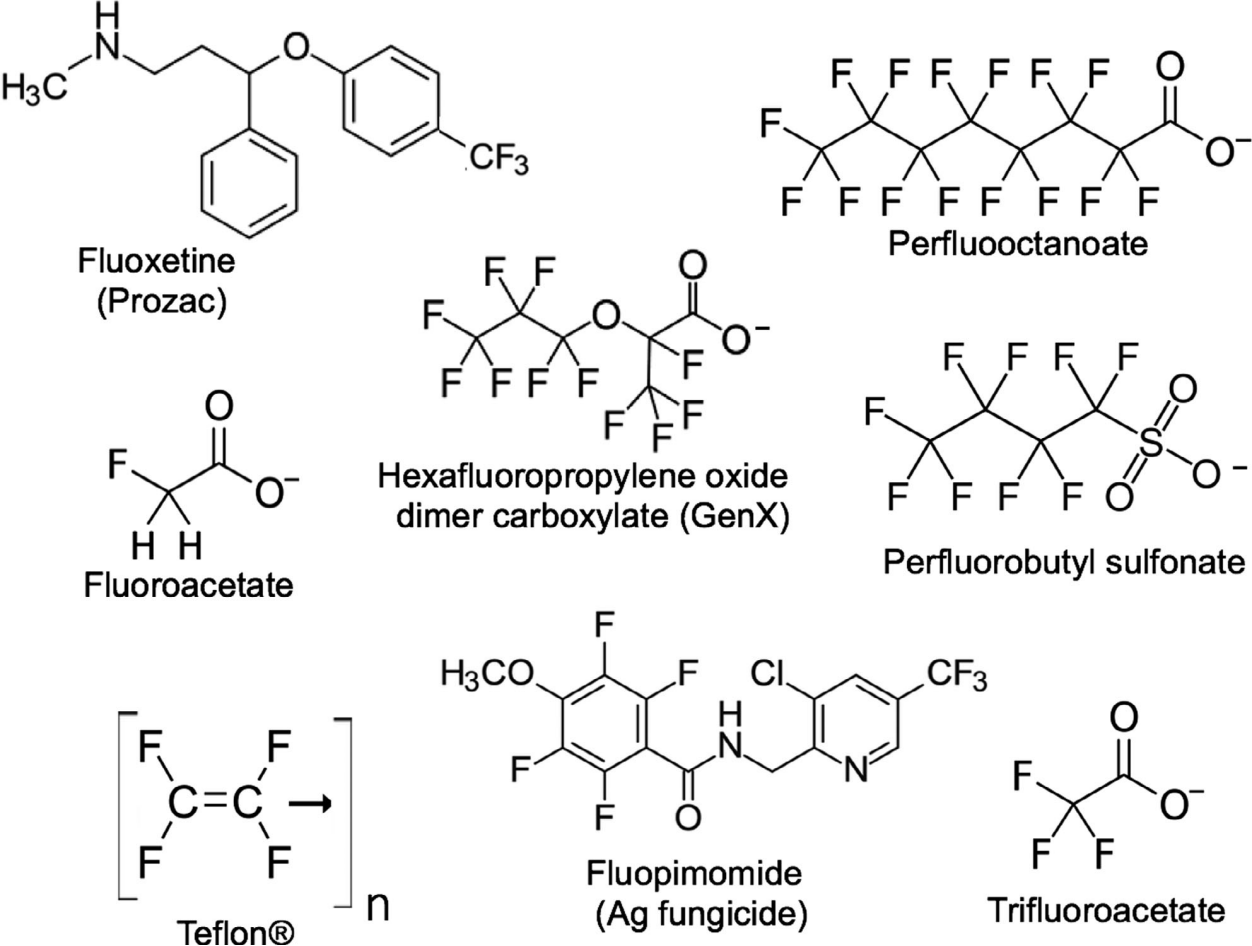
Common fluorinated compounds found in the world. The only natural product, fluoroacetate, has a single fluorine substituent. The others are industrial products and are polyfluorinated. Tetrafluoroethene (lower left) is typically not used directly but is polymerized to make the non‐stick polymer Teflon^®^.

Greater than 9000 heavily fluorinated chemicals have been synthesized for commercial applications (Hogue, 2021). The term ‘perfluorinated alkyl substances’ (PFAS) has become pervasive, but many environmentally relevant fluorinated compounds contain carbon atoms bonded to elements other than fluorine, and so they are best termed polyfluorinated. This review will cover polyfluorinated compounds, both aliphatic and aromatic (Fig. 1). It will discuss perfluorinated compounds and compounds with perfluorinated alkyl groups bonded to other functional groups. While fluoroacetate is not a polyfluorinated compound, it is discussed here because it is the most well‐studied compound with respect to biodefluorination. The knowledge gained from those studies is important to inform ongoing studies with polyfluorinated compounds. The industrial uses of polyfluorinated compounds and moieties have expanded exponentially, being used in more than 200 distinct applications (Glüge *et al*., 2020). The goal is to present microbial successes and failures in biodegrading polyfluorinated compounds and the underlying reasons for those outcomes. It is important to better understand the chemistry, microbiology and evolutionary history underlying the many failures in order that we may better find and, optimistically, engineer successes.

Polyfluorinated compounds are found in every corner of earth, and human and ecosystem toxicity are now well documented (Ahrens and Bundschuh, 2014; Sunderland *et al*., 2019; Sinclair *et al*., 2020; Brase *et al*., 2021; Podder *et al*., 2021; Rice *et al*., 2021). Most recently, negative impact of PFAS exposure includes more severe disease progression following COVID‐19 infection (Grandjean *et al*., 2020). Environmental remediation efforts are increasing exponentially, with billions of dollars being earmarked for the remediation and litigation of polyfluorinated chemicals (Gardella, 2020; Chesler *et al*., 2021; Cordner *et al*., 2021; Lim, 2021). However, designating heavily fluorinated compounds as ‘forever’ chemicals has hindered progress on effective remediation strategies. The term signals to the public, regulators and environmental engineers that nature will never destroy them a priori. This review takes the stance that it is premature to preclude bioremediation of polyfluorinated compounds. Biodegradation removes chemicals permanently and is a relatively inexpensive option for eliminating low concentrations of widely distributed pollutants (Alexander, 1999; Wackett and Hershberger, 2001; Parales *et al*., 2002; Dvořák *et al*., 2017). In that context, bioremediation is often the method of choice for gasoline spills, groundwater plumes of chlorinated solvents and general industrial waste captured in holding ponds.

Fluorinated compounds pose a new challenge to microbial metabolism and hence to the use of microbes for bioremediation. There is a need for more fundamental research on how microbes interact and react with organic and inorganic fluorine. Fluorine differs from the other halogen elements and chemical functional groups in many of its properties (Aigueperse *et al*., 2000; Clark, 2002; Jeschke, 2004; Kirsch, 2004; Emsley, 2011). This review will highlight the uniqueness of organofluorine chemistry and consider microbial metabolism in light of that. Overall, the microbial metabolic degradation of heavily fluorinated compounds is a rare phenotype, but one that must be studied at a fundamental level in order to identify, and hopefully engineer, microbes for more rapid biodegradation. Those doing enrichments or molecular engineering will equally benefit from a background understanding of fluorine chemical reactivity, interaction of organofluorine with biomolecules, a natural history of fluorinated compounds in the environment and the potential toxicity of metabolic products of defluorination metabolism. This contribution will review the published literature on fluorine chemistry and biology with the goal of stimulating efforts to better understand and implement PFAS biodegradation.

## Polyfluorinated, including perfluorinated, compounds are biodegradable

While news reports continue to describe polyfluorinated compounds as ‘forever’ chemicals, the scientific literature details the biodegradation of multiple fluorinated chemicals, including PFAS. Specifically, two recent papers in *Environmental Science and Technology* describe a single bacterium and a consortium, respectively, that catalyze defluorination of PFAS (Huang and Jaffé, 2019; Yu *et al*., 2020). In Huang and Jaffé (2019), microbial incubations with perfluooctanoic acid (PFOA) and perfluorooctanesulfonic acid (PFOS) led to their disappearance concurrent with fluoride anion release, and proper controls were performed to rule out artifacts. Both studies identified defluorinated products derived from the parent compound. Huang and Jaffé showed defluorination concomitant with a progressive shortening of the molecule, with perfluorooctanoate proceeding to perfluoroheptanoate and to perfluorohexanoate and so on (Huang and Jaffé, 2019). A similar degradative series has been demonstrated in chemical studies using strong oxidants or reductants, with proposed radical intermediates causing decarboxylation and subsequent defluorination (Singh *et al*., 2019; Su *et al*., 2019; Filho and Souza, 2020; Liu *et al*., 2021; Palma *et al*., 2021). Given that radical decarboxylation reactions are well known in biological systems (Liu *et al*., 2018, 2018,2018, 2018; Michlits *et al*., 2020; Rodrigues *et al*., 2020; Pastore *et al*., 2021), a radical‐based mechanism for perfluorooctanoate was proposed (Wackett and Robinson, 2020). Moreover, radical desulfonation enzymology has been described (Peck *et al*., 2019; Dawson *et al*., 2021), and radical desulfonation chemistry for PFAS has been described (Niu *et al*., 2016). Another major class of polyfluorinated compounds are the fluoroteleomers, which typically consists of a polyfluorinated chain bonded to several methylene carbon atoms (Kwiatkowski *et al*., 2020). In one recent example, 6:2 fluoroteleomer sulfonic acid is biodegraded via attack at the carbon to sulfur bond to produce a fluoroteleomer aldehyde, oxidized to a carboxylic acid, and subsequently defluorinated by HF elimination from the carbon atoms alpha and beta to the carboxylic acid group (Shaw *et al*., 2019).

Collectively, these studies on the microbiology, enzyme biochemistry and chemistry of polyfluorinated chemicals demonstrate that they are chemically degradable *and* biodegradable. Indeed, a large industry focusing on remediation has arisen using physical and chemical methods (Batelle, 2021; Cordner *et al*., 2021; Envirogen Technologies, 2021; Lim, 2021). Incineration has been investigated, but it is energy intensive for low concentrations in water and may produce hazardous hydrogen fluoride in the off gases (Winchell *et al*., 2021). In addition, electrochemistry, plasma arc technology, and chemical oxidants and reductants are being employed (Singh *et al*., 2019; Su *et al*., 2019; Filho and de Souza, 2020; Liu *et al*., 2021; Palma *et al*., 2021). However, the major drawback to these methods are their non‐specificity, expense and the chances for producing toxic, reactive compounds as byproducts. In this context, reverse osmosis and nanofiltration have been emerging as important water treatment methods (Mastropietro *et al*., 2021). Overall, the most common method for water remediation of polyfluorinated compounds is surface adsorption. Activated carbon is often used because of its relative low cost and familiarity to the water treatment communities (Belkouteb *et al*., 2020; Liu *et al*., 2020; Sonmez *et al*., 2021). Carbon filtration is known to remove many organic contaminants, often present at higher concentration than the fluorinated compounds, resulting in competition for binding sites on the adsorbent. For this reason, there have been accelerated efforts to produce more specific adsorbents, and new companies have emerged from these technologies (Ching *et al*., 2020; Maga *et al*., 2021).

Biodegradation of fluorinated compounds, as observed to date, occurs on scales of weeks and months, the range of chemicals degraded is limited, and there are few enzymes yet identified that represent the different chemical mechanisms of defluorination (Bondar *et al*., 1998; Hasan *et al*., 2011; Tiedt *et al*., 2016; Huang and Jaffé, 2019; Yu *et al*., 2020). A rare example is the enzyme fluoroacetate dehalogenase, which has been studied structurally and mechanistically in significant detail (Goldman, 1965; Kurihara *et al*., 2003; Donnelly and Murphy, 2009; Chan *et al*., 2011; Chan *et al*., 2011; Nakayama *et al*., 2012)). However, the reaction is likely not representative of how polyfluorinated compounds are biodegraded. Difluoroacetate has been found to be much more recalcitrant to biodegradation (Alexandrino *et al*., 2018), while dichloroacetate is readily biodegraded (Slater *et al*., 1985; Busto *et al*., 1992; Thomas *et al*., 1992; Blackburn *et al*., 2000; Dixon *et al*., 2000; Pandey *et al*., 2017; Chen *et al*., 2021). Recently, the enzymatic defluorination of difluoracetate and 2,3,3,3‐tetrafluoropropionic acid has been reported (Li *et al*., 2019; Yue *et al*., 2021). While the lesser fluorinated analogs are not major pollution problems, trifluoroacetate is used in larger scale industrially, and bioremediation systems have been sought, such as in peptide synthesis companies where this strong acid is used in deblocking chemistry (Sakakibara and Inukai, 1965; Pearson *et al*., 1989). Interestingly, the biodegradation of trifluoroacetate was initially reported in 1994 (Visscher *et al*., 1994). However, that enrichment has not been made available, and all efforts to reproduce that result have been unsuccessful till date (Ochoa‐Herrera *et al*., 2016; Alexandrino *et al*., 2018; Yu *et al*., 2020). The report by Huang and Jaffé showed the formation and disappearance of perfluorobutyrate as an intermediate (Huang and Jaffé, 2019), but biodegradation of trifluoacetate was not examined in that study.

In total, the evidence that fluorinated compounds, including polyfluorinated compounds, are biodegraded is compelling. However, it is also evident that this metabolism is confined to a small number of microbial cultures. In addition, when it does occur, the rates of defluorination of heavily fluorinated compounds is low. The probable reasons for the rarity and modest rates of biodegradation are discussed below.

## In biology, fluorine is very rare while chlorine, bromine and iodide are much more prevalent

**Fig. 2:**
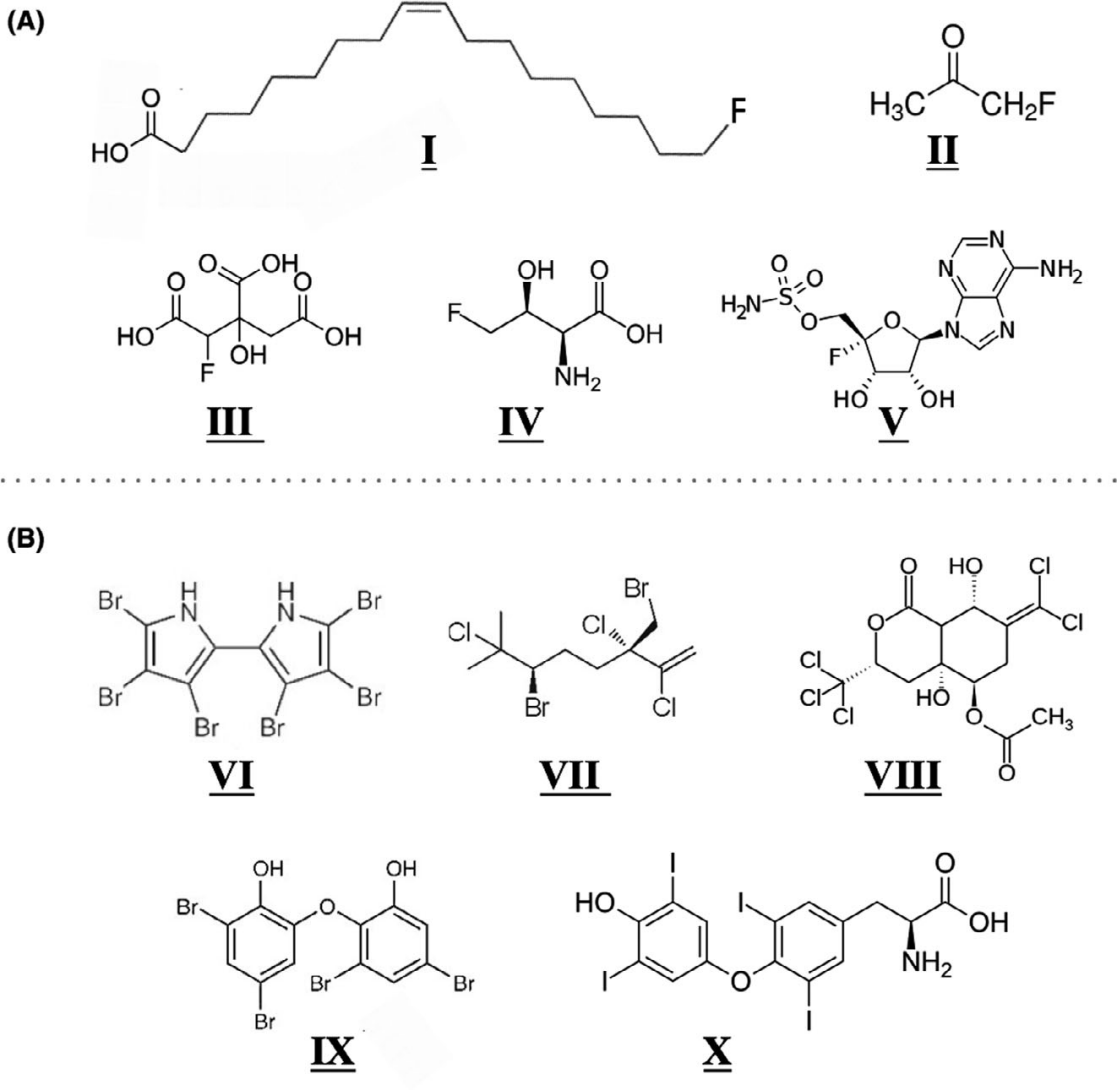
Comparing and contrasting (A) fluorinated natural products (NPs) and (B) chlorinated, brominated and iodinated NPs. A. Fluorinated NPs have only a single fluorine substituent and are typically analogs of biomolecules: **I** is 18‐fluorosteric acid, **II** is fluoroacetone, **III** is fluorocitric acid, **IV** is fluorothreonine, **V** is nucleocidin. B. NPs Cl, Br, and I may contain multiple halogen substituents, examples with 4–6 per molecule are shown. **VI**, **VII**, and **IX** are marine natural products. **VIII** is sigillin A, a deterrent produced by a flea. **X** is the human hormone thyroxine.

The halogen elements on earth are largely combined with other elements and in that largely inorganic form, fluorine is the most abundant of the halogens on Earth and is overall the 13^th^ most abundant element (Allegre *et al*., 1995; McDonough, 2021). Despite that, fluorine is relatively insignificant biologically, and this contrasts with the halogens chlorine, bromine and iodine. Cellular chloride transport is an important physiological function in bacteria, plants and mammals (Chen, 2005). The genomes of many bacteria and archaea encode a Clc‐type chloride channel to import and maintain chloride balance (Iyer *et al*., 2002). In *Escherichia coli*, a mutation in the chloride channel showed it to be essential for pH homeostasis and acid stress survival (Maduke *et al*., 1999). Thousands of chlorinated natural products have been identified over the last few decades (Fig. 2) (Gribble, 2012, 2015; Dong *et al*., 2020; Ludewig *et al*., 2020), and brominated natural products are common in marine environments (Küpper and Carrano, 2019). Certain algae accumulate high levels of iodine (Mondal *et al*., 2021), and iodine represents more than half the mass of the human hormone thyroxine (Thomas *et al*., 2009). Indeed, many chlorinated, brominated and iodinated natural products contain four or more halogen substituents (Fig. 2). Multiple enzymes have been identified that produce organohalides, largely containing chlorine, bromine or iodine. In fact, six different classes of halogenase enzymes are known (Walker and Chang, 2014; Agarwal *et al*., 2017; Murphy *et al*., 2017; Fejzagić *et al*., 2019; Liu *et al*., 2021). Halogenase activity has apparently evolved independently from different protein folds and utilizing different (or no) cofactors. The classes are as follows: (1) heme‐iron haloperoxidases, (2) non‐heme iron halogenases, (3) flavin halogenases, (4) S‐adenosyl‐l‐methionine halogenases, (5) vanadium haloperoxidases and (6) cofactor‐free haloperoxidases.

By contrast, bacteria and other organisms do not employ fluorine as they do chlorine and other halogens. Fluoride is not accumulated. In fact, it is highly toxic to bacteria, as discussed in a later section. Moreover, biosynthesis of organofluorides by all living things is rare (Murphy *et al*., 2003; Fincker and Spormann, 2017). The naturally occurring organofluorine compounds are singly fluorinated (Fig. 2) and are typically analogs of common metabolites, such as acetate, citrate, amino acids and nucleotides. This contrasts with chlorinated and brominated natural products, which are often unique structures and may contain a large number of halogen substituents.

It is hypothesized here that the paucity of natural fluorinated compounds compared to other organohalides is one factor underlying the rarity of PFAS biodegradation. It is well accepted that dehalogenases reactive with organochlorine and organobromine compounds have an ancient evolutionary origin and processed natural products prior to industrialization (Leisinger *et al*., 1994; Valverde *et al*., 2004; Quack *et al*., 2007; Futagami *et al*., 2008; Liang *et al*., 2012; Hug *et al*., 2013; Richardson, 2013; Jugder *et al*., 2016). Some aromatic natural products substituted with chlorine and bromine substituents (Fig. 2) resemble specific industrial chlorinated biphenyl and brominated aromatic ethers (Hou *et al*., 2021). Polyhalogenated methanes are also natural products (Gribble, 2015) and may have primed the biodegradation of industrial halomethane solvents. For example, a bacterium isolated from a bioreactor degrading dichloromethane contains a dehalogenase that produces formaldehyde, which feeds into the organism’s C_1_ metabolic pathways. The enzyme dichloromethane dehalogenase is found broadly with widely divergent sequences, consistent with a long evolutionary trajectory. Moreover, it may have evolved under selection for metabolizing the natural product dibromomethane (Quack *et al*., 2007). The enzyme has a higher *k*_cat_ and a lower K_M_ for dibromomethane than dichloromethane (Scholtz *et al*., 1998). In contrast, there is evidence for newly evolved dehalogenases that act on anthropogenic organochloride pesticides (Wackett, 2004; Copley, 2009). Two example enzymes for which there is good evidence for recent evolution are atrazine chlorohydrolase (Seffernick and Wackett, 2001) and 1,3‐dichloropropene dehalogenase (Poelarends and Whitman, 2004).

Fluorine‐containing organic compounds are completely different. There are on the order of a dozen fluorine‐containing natural products and well over one million synthetic organic compounds containing fluorine, as found in a search of PubChem (Kim *et al*., 2021). The synthetic compounds have only entered commerce, and the environment, in the last few decades. These new organofluorine compounds have been thrust upon relatively unprepared microbial populations. This contrasts with the earlier explosion in synthesis of organochloride commercial chemicals in the 1930s and 1940s that met a microbial population exposed to highly chlorinated natural products for millions of years (Fig. 2). Clearly, microbes are at an evolutionary disadvantage with respect to PFAS and other fluorinated organic chemicals.

## Fluorine chemistry is different from other halogens, fluorine microbiology must differ too

Overlaid on this evolutionary consideration are the chemical impediments to the biological disposition of organofluorides, which will be discussed here. However, only a relatively cursory treatment of organofluorine chemistry will be presented, and the reader is recommended to consult more detailed and excellent reviews published elsewhere (Kissa, 2001; Smart, 2001; Dolbier, 2005; O'Hagan, 2008; Hügel and Jackson, 2012).

In the microbiological literature, the rarity of organofluorine degradation is often attributed to the C–F bond being chemically much harder to cleave than other C‐halogen bonds in comparable compounds. This is an oversimplification. Indeed, it is not universally correct because in nucleophilic aromatic substitution reactions, the C–F bond is more readily cleaved than comparable C–Cl and C–Br bonds. That knowledge underlies the use of 1‐fluoro‐2,4‐nitrobenzene, or Sanger’s reagent, for reaction with, and development of a detection method for, amino acids (Sanger, 1945). In addition, as previously discussed, many bacteria produce a fluoroacetate dehalogenase that hydrolyzes a natural product in which the single C–F bond is reasonably reactive, activated by the adjacent carboxylate moiety. This review article seeks to describe the features of C–F chemistry that most relate to the variable biodegradability of different organofluorines as it pertains to their structures and individual reactivities.

While it is difficult to completely generalize, some chemical properties of fluorine and organofluorine compounds can be described (Table 1). Fluorine is the most electronegative atom, which is why organofluoride degradation invariably leads to the electrons leaving with the fluorine atom as an anion. The small size of fluorine is important in the use of fluorine in drug development, since fluorine is not too much larger than a hydrogen substituent and can often substitute sterically without compromising target binding. Such a substitution can serve to substantially moderate drug clearance, an important consideration in drug efficacy (Meanwell, 2018; Al‐Harthy *et al*., 2020; Hevey, 2021; Richardson, 2021). The lower reactivity of designed organofluorine drugs with Phase I drug metabolism enzymes, typically oxygenases, is paralleled by a typically lesser biodegradation by bacterial oxygenases that act on hydrocarbon substrates in the environment. This has resulted in a recent trend of an increased incorporation of fluorine into agrichemicals to increase environmental lifetime and efficacy (Jeschke, 2017; Burriss *et al*., 2018; Ogawa *et al*., 2020). Additionally, fluoride anions interact much more strongly with water compared to other halide anions and this might partially explain why biological systems have rarely incorporated fluoride into biomolecules (Smart, 2001; Tahaikt *et al*., 2007). This also relates to fluoride toxicity to cells, which will be discussed later.

**Table 1:** Chemical properties of fluorine relevant to microbial physiology.

General
Highest electronegativity by far
Smaller than any carbon substituent after hydrogen
Fluoride ion is highly solvated in water
Forms tight bonds with carbon, wide variability in bond strength
Low polarizability
In partially fluorinated molecules
Overall molecule often very polar
Variable reactivity
In perfluorocarbons (compounds with only C–C and C–F bonds)
Often lower boiling than the analogous hydrocarbons of much lower MW
High vapor pressures
Poorly soluble in water and organic solvents
Poorly reactive with many reagents

The carbon‐to‐fluorine bond strength is often reported as the strongest known bond, but the variation in bond strength is considerable (Table 1). For example, the bond strength for CF_3_–F is 130.5 kcal/mol whereas CH_3_–F is 108.3 kcal/mol. Another literature source reports a C–F bond strength of 154 kcal/mol in hexafluorobenzene (Edelbach and Jones, 1997). Yet, as previously discussed, molecules such as fluoroacetate (Goldman, 1965; Nakayama *et al*., 2012) and various fluoronitrobenzenes (Zhao *et al*., 2014; Xu *et al*., 2019) are relatively reactive, or can be activated, and are readily biodegraded by microbes, so the C–F bond is not uniformly recalcitrant to microbial disruption. Moreover, bond strength is not the sole determinant of reactivity in organofluorine chemical reactions. For example, while C–F bonds are generally stronger than C–H bonds in aromatic and aliphatic compounds containing both, chemists have learned to selectively cleave either bond using metallo‐catalysts (Eisenstein *et al*., 2017). Selective C–F cleavage takes advantage of bond cleavage mechanisms in which the more exothermic fluoride forming reaction is favored. In general, aryl and olefinic fluorine compounds are more reactive than perfluoroalkanes since the π‐bonds are subject to nucleophilic attack and fluoride is a good leaving group in metal‐catalyzed reactions of this type (Kiplinger *et al*., 1994). Defluorination of fluoroolefins with replacement by carbon dioxide was shown to be carried out in good yield by copper catalysts (Gao *et al*., 2020). Recently, a perfluoroalkenoic acid was shown to undergo defluorination at an olefinic carbon by a microbial consortium (Yu *et al*., 2020).

Perfluoroalkanes are generally less reactive, but are not completely inert and are capable of undergoing reductive defluorination (Saunders, 1996). However, reduction of non‐branched fluorocarbons containing only secondary and primary C‐F bonds is rare. This is due to the low reduction potentials, generally less than −2.7 volts (V) (Park *et al*., 2009).

**Fig. 3:**
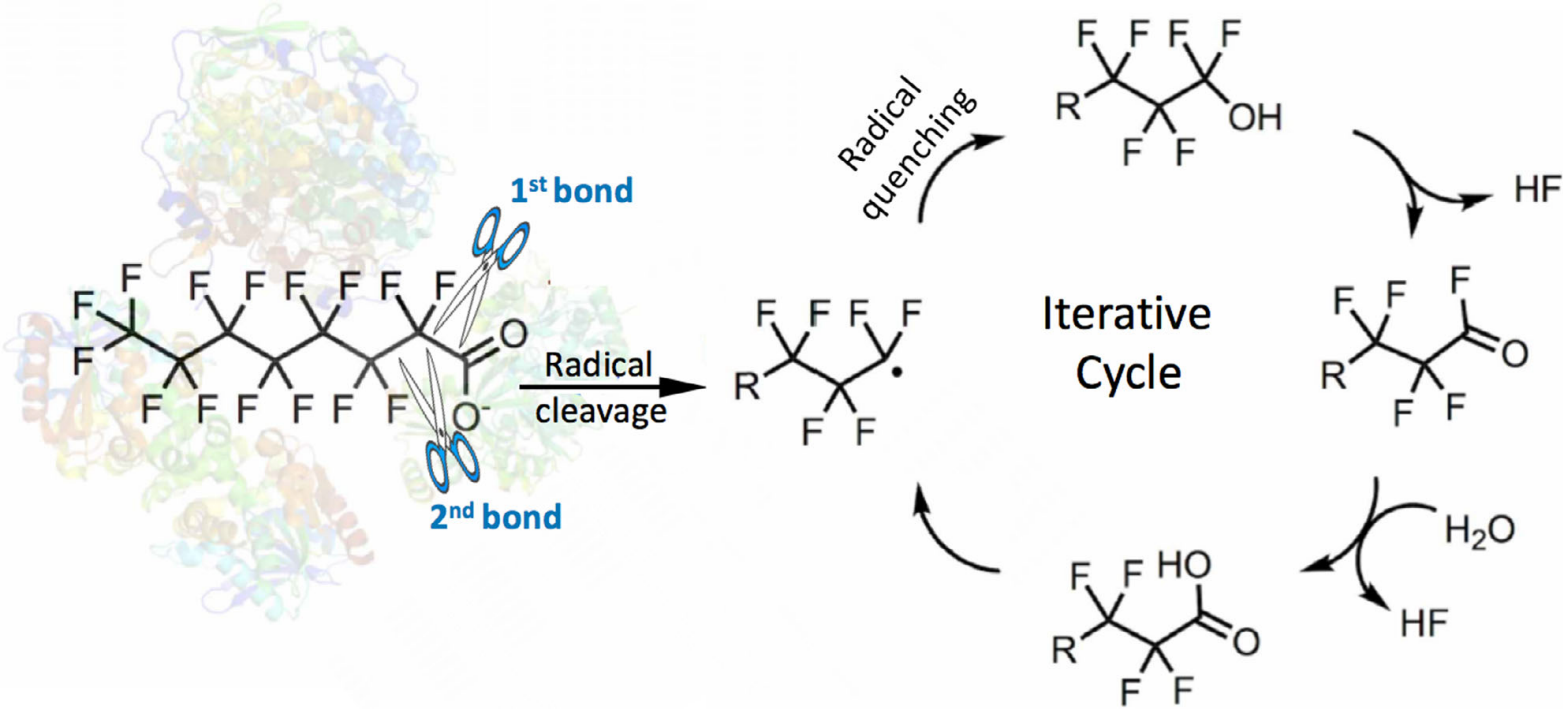
A proposed mechanism for the complete defluorination of perfluorooctanoate. The mechanism shows initiating cleavage of the C_1_‐C_2_ bond in a radical fashion and quenching of the radical to make a C_7_ pefluorinated alcohol. The reactions of the alcohol to the carboxylic acid on the right are non‐enzymatic and rapid. The resultant C_7_ carboxylic acid can undergo the same reaction cycle to make a C_6_ carboxylate, and then shorter chains, iteratively.

Fortunately, most commercially important PFAS are not perfluoroalkanes, but contain other functional groups. Two prominent examples are perfluorooctanoic acid (PFOA), perfluorooctanesulfonic acid (PFOS) and derivatives thereof (Wang *et al*., 2019). In addition to C–F bonds, these molecules contain C–C(carboxylate) and C–S(sulfonate) bonds that represent another weaker point of attack. Indeed, one‐electron reduction or oxidation of these bonds to generate a carbon‐centered radical, with subsequent installation of a hydroxyl group, is plausible (Fig. 3). This will generate a shorter chain by one via facile *gem*‐elimination and hydrolysis reactions, and that cycle will repeat (Fig. 3). That is a plausible biodegradation mechanism, and there is precedent for this with non‐biological oxidative and reductive chemistry as has been demonstrated in numerous publications (Park *et al*., 2009; Liu *et al*., 2021; Palma *et al*., 2021).

Another distinctive chemical feature of fluorine in organic molecules has relevance to the availability and binding of these chemicals in biological systems. Perfluorocarbons have very low water solubility (Dalvi and Rossky, 2010). In fact, perfluorocarbons are poorly soluble in organic solvents and thus can form a third ‘fluorous phase’ when added to a two‐phase water/organic solvent mixture (Studer *et al*., 1997; Dobbs and Kimberley, 2002). Hydrofluorocarbons are much more water soluble and perfluoro carboxylate and sulfonate compounds show greatly variable solubility depending upon their carbon chain length. With longer chain lengths, water solubility decreases. PFAS compounds in natural waters, where their concentrations are typically parts per billion or parts per trillion, will be dissolved.

Although solubilities can be measured directly, much less is known about how PFAS compounds such as PFOA and PFOS might move out of an aqueous environment and into bacterial cells. There is evidence that these compounds can partition into cellular membranes passively (Fitzgerald *et al*., 2018b); in one case, this was shown to affect the quorum sensing response (Fitzgerald *et al*., 2018a). It is less clear if these molecules are actively transported, perhaps adventitiously by transporters evolved for other uptake functions.

Fluorinated molecules, even perfluorinated, can bind to non‐catalytic proteins such as bovine serum albumin (Fedorenko *et al*., 2021). This indicates that natural proteins and their amino acid side chains have the potential for evolving pre‐equilibrium binding of these molecules as substrates. Indeed, a recent study has analyzed the human protein ‘interactome’ of perfluorooctanephosphonic acid (PFOPA) and identified 469 PFOPA‐binding proteins (Zhang *et al*., 2021).

## Mechanisms and limitations for microbial organofluorine biodegradation

**Fig. 4:**
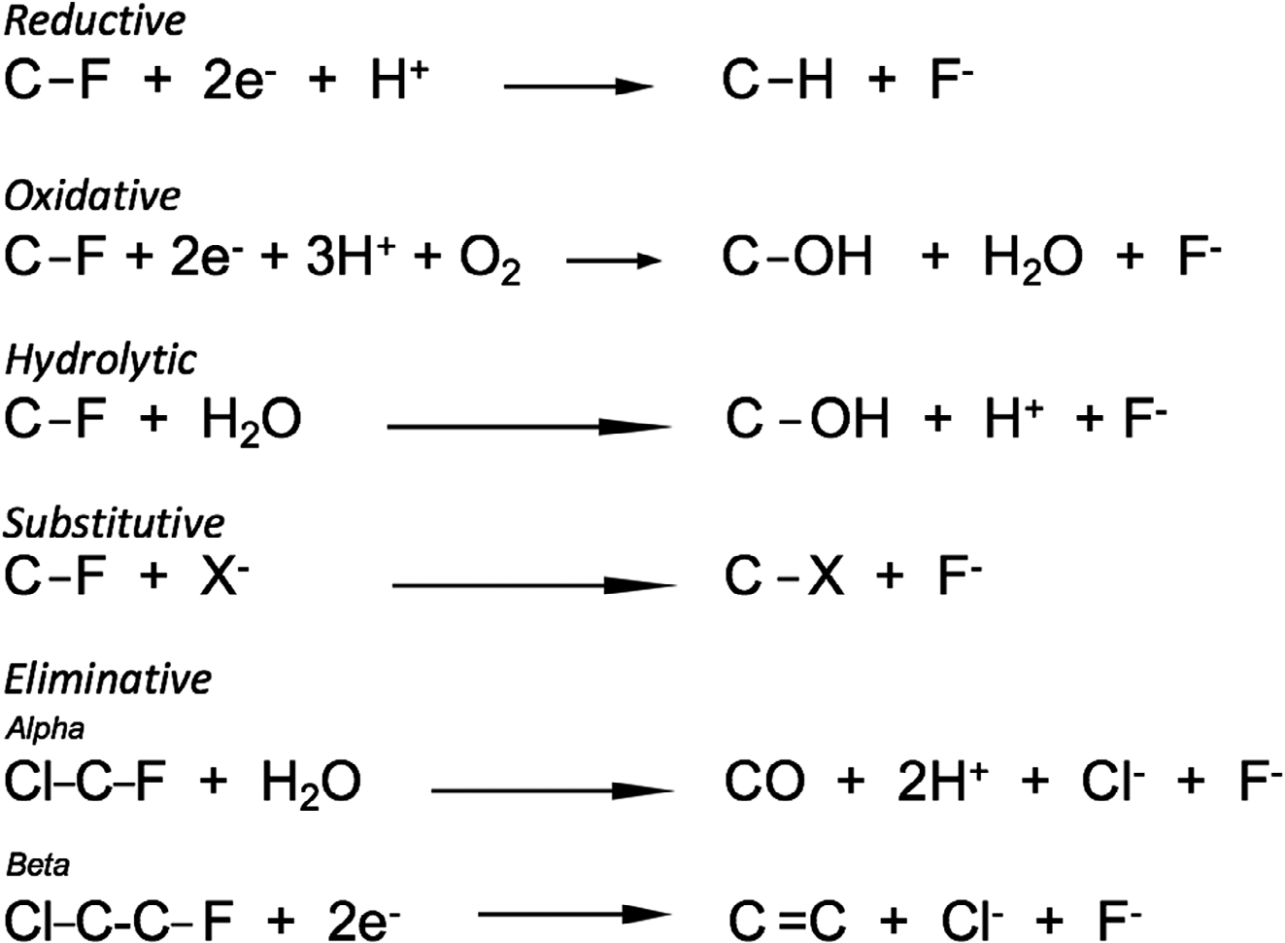
Generalized mechanisms of defluorination based on examples in the scientific literature. The carbon atoms may be alkyl, olefinic or aromatic. The reactions are simplified to show the key elements of each reaction type. *Beta*‐elimination can also occur with H‐C‐C‐F containing compounds and produce H^+^ and F^−^ as products.

Microbial degradation of organochlorides has been extensively studied, and so it is useful to compare analogous chlorinated and fluorinated compounds here. Studies of microbial dechlorination physiology have revealed five general types of reactions: (1) reductive, (2) oxidative, (3) hydrolytic, (4) substitutive, and (5) eliminative (Fig. 4). Based on known chemistry as previously discussed, the propensity for organofluorine biodegradation following each of these mechanisms will be compared and contrasted with organochlorine biodegradation. Since this review focuses on fluorine, C–F bonds are illustrated. However, it should be understood that there are many more examples of C–Cl bond cleavage for each type of reaction. As previously discussed, fluorine differs from other halogens and so we would anticipate that many dechlorinating enzymes would fail to catalyze defluorination of analogous molecules. Exceptions are known with some reasonably reactive monofluorinated compounds such as fluoroacetate (Kurihara *et al*., 2000), fluoroatrazine (Seffernick *et al*., 2000) and 2‐fluorobenzoate (Engesser and Schulte, 1989).

### Reductive defluorination

In the mid‐twentieth century, many chlorinated synthetic pesticides were considered to be highly persistent in the environment, even non‐biodegradable (Wackett and Robinson, 2020). It was realized in the 1960s that certain of these organochloride and organobromide pesticides underwent reductive dehalogenation in soil (Castro and Belser, 1968). Subsequent research showed that reduced metallo‐enzymes and their cofactors could catalyze reductive dechlorination (Stotter *et al*., 1977; Schrauzer and Katz, 1978). Subsequently, chloro‐aromatics (Suflita *et al*., 1982), chloro‐alkenes (Vogel and McCarty, 1985) and chloroalkanes (Gälli and McCarty, 1989) were shown to undergo microbially catalyzed reductive dechlorination (Fig. 4). Enzymes have been purified, structures determined, and reactions shown to be dependent on reduced low‐potential cobalt‐corrinoid cofactors (Neumann *et al*., 1996; Kräutler *et al*., 2003; Payne *et al*., 2015). To this author’s knowledge, none of these well‐characterized dechlorinating and debrominating enzymes have been shown to catalyze reductive defluorination, but reduced corrinoids have been demonstrated to have defluorination activity with polyfluorinated compounds (Ochoa‐Herrera *et al*., 2016; Liu *et al*., 2018, 2018,2018, 2018; Sun *et al*., 2021).

A key feature of reductive dechlorination came to light with the realization that polychlorinated biphenyls (PCBs) and other aromatic and aliphatic compounds can serve as final electron acceptors in respiratory metabolism (Brown *et al*., 1987; Mohn and Tiedje, 1990; Schumacher and Holliger, 1996; Susarla *et al*., 1997; Holliger *et al*., 1998; Smidt *et al*., 2000; Duhamel and Edwards, 2007). Microorganisms will often utilize respiratory over fermentative metabolism, when they are capable of both, to capture more energy. In this context, anaerobes use many different electron acceptors in place of oxygen to maximize energy acquisition. Some bacteria are now known to catalyze reduction of C–Cl bonds in a manner that is analogous to the reduction of nitrate or sulfate. Halo‐respiring organisms couple dechlorination to electron transport‐driven ATP generation (Smidt and de Vos, 2004). This process of organochlorine and organobromine respiration is now considered to be reasonably common, evolutionarily ancient, and further adapted in response to the many chlorinated and brominated natural products produced in the environment (Mayer‐Blackwell *et al*., 2016; Tang *et al*., 2016). The process is thermodynamically feasible and beneficial because the redox potential of the C–Cl bond in many chloroaromatics and chloroaliphatics is in the range of +250 to +600 mV (Holliger *et al*., 1998). So many simple metabolic oxidation reactions, for example, the two‐electron oxidation of succinate to fumarate at +32 mV (Thauer *et al*., 1989), have a lower redox potential and could theoretically couple to chloro‐reduction to input electrons into the respiratory chain and ultimately produce ATP for the organism.

C–F bond reduction is not analogous energetically. As discussed previously under the preceding section, the redox potential of −2700 mV or lower is well below any oxidizable growth substrate for bacteria and out of the range of biological redox carriers. PFAS compounds that are more amenable for reduction, such as PFOS and PFOA, are reported to have redox potentials in the −450 mV range (Park *et al*., 2009). While this is within the low end of redox catalysts in biology, it is much too low for the reduction reaction to be coupled to a respiratory chain and generate ATP for a bacterium. Oxidizable substrates for microbes typically have redox potentials more positive than −450 mV, and so transfer of electron from those substrates to PFAS reduction would be thermodynamically unfavorable. Therefore, unlike C–Cl bond reduction, C–F bond reduction seems unlikely to be coupled to ATP generation.

In light of this, how might the C–F bonds of polyfluorinated compounds with a low but biologically accessible redox range, in the range of −400 to −700 mV, be amenable to reductive defluorination? By way of calibration, the low potential coenzyme for many cellular reduction reactions, NADH, has a standard redox potential of −320 mV and would not directly couple in these reaction. There are, however, several important processes microorganisms have evolved to meet the need for low‐potential reduction metabolism. One is the evolution of enzymatic machinery for the six‐electron reduction of atmospheric dinitrogen to produce two atoms of ammonia, a process known as nitrogen fixation and catalyzed by the enzyme nitrogenase. The redox potential of the nitrogenase Fe–Mo protein is reported to be approximately −540 mV (Milton and Minteer, 2019). Since nitrogen‐fixing bacteria often grow on oxidizable carbon and energy sources with more positive redox potentials than −540 mV, ATP is required to ramp down redox potentials through a series of electron carriers to the Fe‐Mo protein (Rutledge and Tezcan, 2020). Another example of low potential metabolism that has evolved in certain bacteria is represented by enzymes mediating anaerobic growth on benzene‐ring compounds via an initial reduction reaction (Wischgoll *et al*., 2005; Löffler *et al*., 2011). The benzene ring is stabilized by a resonance energy of 36 kcal mol^−1^ over 1,3,5‐hexatriene, so reduction of the benzene ring, as carried out by organic chemists, employs extremely powerful reducing reagents such as elemental sodium in liquid ammonia (Zimmerman, 2012). The bacterium *Geobacter metallireducens* meets this demand by biosynthesizing a one megadalton enzyme complex that can attain a redox potential of −622 mV (Huwiler *et al*., 2019). Like nitrogenase, attainment of a requisite ultra‐low redox potential requires ATP.

Indeed, there is precedence for ATP‐dependent reductive defluorination (Tiedt *et al*., 2016). *Thauera aromatica* has been shown to catalyze an ATP‐dependent reaction in which 4‐fluorobenzoyl‐CoA is overall reductively defluorinated to benzoyl‐CoA and HF. The reaction is catalyzed by an enzyme homologous to known benzoyl‐CoA reductases and is proposed to proceed via an aromatic ring reduction followed by HF elimination. This is an example of defluorination of a monofluorinated compound but, as discussed above, may prove relevant to polyfluorinated compounds.

Accrued knowledge of PFAS redox chemistry, microbiological redox biochemistry and direct microbiological data (Tiedt *et al*., 2016) suggests that C–F bond reduction will require ATP, in contrast to C–Cl bond reduction that can generate ATP. Earlier in this review, evolution was said to favor metabolism of organohalides other than organofluorides because of the prevalence of polychlorinated and polybrominated natural products. Another disadvantage for C–F reduction is that it may be selected against in evolution because it provides no benefit, and may even drain cellular energy. This further explains why the reduction of PFOA and PFOS may be rarely identified and relatively slow, consistent with reports of laboratory persistence (Dinglasan *et al*., 2004; Parsons *et al*., 2008; Murphy, 2010), and a limited number of recent successful enrichments that show relatively slow biodegradation rates (Huang and Jaffé, 2019; Yu *et al*., 2020). It is important to understand the molecular basis of the defluorination reactions to better find natural representatives and to potentially re‐engineer microbial ultra‐low redox systems for PFAS biodegradation. Note that nitrogenase is being re‐engineered in this manner (Milton and Minteer, 2019) and is now demonstrated in the laboratory to generate hydrocarbons (Yang *et al*., 2011) and other organic products (Seefeldt *et al*., 2013) in an effort to extend the enzyme’s catalysis beyond its evolved function of dinitrogen reduction.

### Oxidative defluorination

There are numerous reports of oxidative defluorination, most of which involve aromatic or other ring systems with single fluorine substituents. Excellent review articles are available covering C‐F bond cleavage with a focus on aromatics (Kiel and Engesser, 2015) and reactions catalyzed by metalloenzymes (Wang and Liu, 2020). Many of the metalloenzymes catalyzing defluorination are oxygenases, oxidases and peroxidases. The mechanisms of monodefluorination have been studied with cytochrome P450 monooxygenases (Harkey *et al*., 2012), dioxygenases (Li et al., 2020; Renganathan, 1989) and oxidases (Li *et al*., 2020). Recently, bacterial degradation of monofluorinated alkyl chains via oxygenase‐initiated reactions was reported (Xie *et al*., 2020). This type of reaction is best represented by the second mechanism from the top depicted in Fig. 4.

Bacterial oxidative biodegradation of polyfluorinated olefins is relatively rare. An exception is the biodegradative defluorination of trifluoroethylene by the soluble methane monooxygenase from *Methylosinus trichosporium* OB3b (Fox *et al*., 1990). The major product of the reaction was glyoxylate. The rate of oxidation was only 10% of the rate with trichloroethylene. Chlorotrifluoroethylene was oxidized to oxalate, consistent with complete dehalogenation, but the rate was only 25% of that for trifluoroethylene.

The major focus on this review article is on polyfluorinated compounds, particularly aliphatic compounds with carbon atoms containing multiple fluorine substituents. In drug design, it is widely appreciated that substituting trifluoromethyl for methyl groups will block oxygenases that initiate metabolism and thus slow drug clearance (Hagmann, 2008; Sun *et al*., 2011). Trifluoromethyl groups on aromatic rings often persist, even as the ring may be oxidized, and the three C‐F bonds can end up on trifluorocetate as a metabolic end‐product (Key *et al*., 1997; Kiel and Engesser, 2015). Difluoromethyl substituents are much less common in commercial compounds. They have been examined as anti‐metabolites and inhibitors in medical chemistry. A difluoromethyl‐analog of progesterone has been suggested to be hydroxylated at the –CHF_2_ group due to its effectiveness as an irreversible inhibitor. Inhibition is proposed to result from hydroxylation, *gem*‐elimination and reaction of the resultant acyl fluoride with the cytochrome P450, thus inhibiting the enzyme (Stevens, 1991).

### Hydrolytic defluorination

The prototypic bacterial enzyme catalyzing hydrolytic defluorination is fluoroacetate dehalogenase. As previously discussed, fluoroacetate (Fig. 1) is a natural product and highly toxic, so bacteria may benefit from detoxification and acquisition of carbon and energy from the defluorinated product, glycollate (Goldman, 1965). In this context, fluoroacetate is degraded by bacteria isolated from the rumen of cows, which may periodically ingest one or more of the forty plants known to biosynthesize fluoroacetate (Davis *et al*., 2012). Fluoroacetate dehalogenase enzymes have also been identified in diverse soil bacteria of the genera *Moraxella, Delftia, Burkholderia, Pseudomonas* and *Rhodopseudomonas* (Seong *et al*., 2019). Many of these enzymes isolated as fluoroacetate dehalogenases have some activity with chloroacetate and bromoacetate. However, fluoroacetate typically shows the highest catalytic efficiency, *k*_cat_/*K*_M_, with enzymes designated as fluoroacetate dehalogenases.

The nature of the relative catalytic efficiency with fluoroacetate has been most well‐studied with the enzyme denoted FacD from *Burkholderia* sp. FA1 via studies involving X‐ray crystallography, site‐directed mutagenesis, rapid synchotron crystallography and computational studies (Chan *et al*., 2011; Miranda Rojas *et al*., 2018; Schulz *et al*., 2018). The reaction is known to proceed via nucleophilic attack of an aspartate nucleophile to generate an aspartate ester intermediate that is resolved by attack of water activated by an active site histidine. This is a fairly unremarkable mechanism common to many enzymes with a general α/β hydrolase fold. Rather a major factor in reduction of the activation barrier of C‐F bond cleavage is the specific interaction of the fluorine with the halogen pocket. The halogen pocket is defined by the triad of a tryptophan, histidine and tyrosine within a distance of 3.0 to 3.3 Angstrom from the fluorine that defines a fluorine‐specific pocket in the enzyme–substrate complex. Computational studies have further defined optimum bond angles and plausible energy barriers to facilitate C‐F bond cleavage.

### Substitutive defluorination

Rumen bacteria with hydrolytic fluoroacetate dehalogenases are known to protect animals that have ingested fluoroacetate‐containing plant material (Loh *et al*., 2020). However, fluoroacetate is also detoxified by enzymes that substitute atoms for fluorine other than the oxygen of a water molecule, with the nucelophile represented as X^−^ in Fig. 4. These types of reactions have been most studied in plants and animals that use their own enzymes for detoxification. An enzyme was identified in mammals that catalyzed the substitution of the fluorine atom of fluoroacetate with the sulfur atom of the tripeptide glutathione (Soiefer and Kostyniak, 1983). The enzymes, later known as *theta*‐glutathione‐*S*‐transferases, are found in non‐mammalian sources as well, so the ability to detoxify fluoroacetate may be found broadly throughout biological systems (Sheehan *et al*., 2001). Interestingly, an enzyme of the same class is thought to catalyze defluorination of the difluoromethylene moiety of the anesthetic methoxyflurane (Wang *et al*., 1986).

### Eliminative defluorination

The classic eliminative dehalogenation reaction is that catalyzed by the LinA dehydrohalogenase. LinA transforms the pesticide lindane, or gamma‐hexachlorocyclohexane, to 1,3(R),4(S),5(S),6(R)‐pentachlorocyclohexane via the elimination of H^+^ and Cl^−^ from adjacent carbon atoms, similar to the *beta*‐elimination of two halide substituents shown in Fig. 4. The reaction has been studied in some detail (Nagata *et al*., 2001; Manna *et al*., 2015). Analogous dehydrofluorination reactions have been shown to occur with enzymes in which synthetic fluorinated substrate analogs have been used as mechanistic probes (O'Hagan and Rzepa, 1997; Gulick *et al*., 2001). Dehydrofluorination of polyfluoroalkanes via non‐enzymatic catalysis is known (Pedler *et al*., 1972; Li *et al*., 1998).

There are examples of fluoride eliminations that occur non‐enzymatically after attack by reductive (Tiedt *et al*., 2017) or oxygenative (Fox *et al*., 1990) enzymes, as previously discussed. A somewhat novel *alpha*‐elimination was observed following reduction of fluorotrichloromethane by a bacterial cytochrome P450 followed by chloride elimination, fluorchlorocarbene formation, and then loss of both remaining halide ions (Fig. 4) (Li and Wackett, 1993). Most recently, a *beta*‐elimination pathway was proposed for the defluorination and complete biodegradation of 3,3,3‐trifluoropropionic acid by a sludge sample from a wastewater treatment plant (Che *et al*., 2021).

## Defluorination produces fluoride, and fluoride is highly toxic to all living things

It is proposed here that there is another very significant reason why so few microbes have evolved the capabilities to biosynthesize or biodegrade organofluoride compounds. With fluoride being the most electronegative element, the C–F bond is invariably cleaved heterologously to yield fluoride. However, fluoride is highly toxic to prokaryotic and eukaryotic cells.–Fluoride and chloride are very different environmentally and biologically. While fluorine is significant in the earth’s crust, it is largely bound up in minerals. The ratio of chloride to fluoride in seawater is > 7600 to 1 (Greenhalgh and Riley, 1961). Chloride, is also much more prevalent in terrestrial water sources, where it can be found at > 4 M in some saline seas. Halophilic bacteria survive and thrive in 4 M chloride concentrations in these environments (Müller and Oren, 2003). Bacteria growing in lower saline environments also use chloride extensively. For example, the light‐driven chloride pump proteins classified as rhodopsins have been extensively studied (Inoue *et al*., 2015). Some bacteria have been found to contain ~ 1 M chloride intracellularly (Chen, 2005).

Fluoride is not only much less prevalent in natural waters, but it is also highly toxic to organisms in general. The World Health Organization has recommended a human limit for fluoride in drinking water to be 1.5 mg l^−1^, or 79 μM (Edmunds and Smedley, 2013). In certain waters, fluoride concentrations as low as 26 μM can adversely affect some invertebrates and fishes (Camargo, 2003). Fluoride is also highly toxic to bacteria. This has been exceptionally well documented in many different types of experiments with diverse bacteria (Bhatnagar and Bhatnagar, 2004; Adamek *et al*., 2005; Ochoa‐Herrera *et al*., 2009; Baker *et al*., 2012; Breaker, 2012; Stockbridge *et al*., 2012; Ji *et al*., 2014; Nelson *et al*., 2015; Liao *et al*., 2017; Liu *et al*., 2017; Last *et al*., 2018; Turman *et al*., 2018; Zhu *et al*., 2019; Johnston and Strobel, 2020; Dionizio *et al*., 2021).

**Fig. 5:**
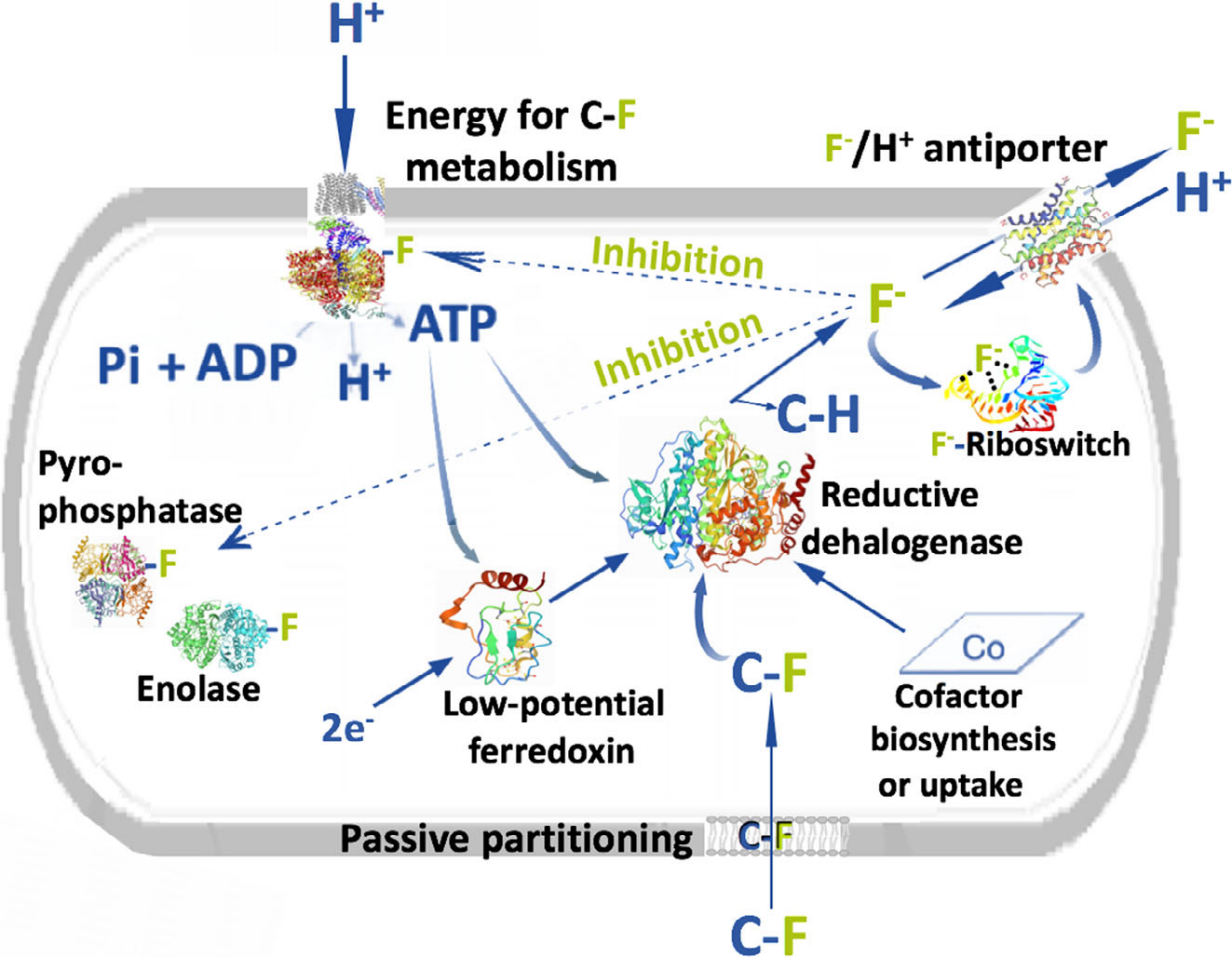
Visualizing requirements of a bacterial cell found naturally, or via laboratory engineering, that would be capable of rapid defluorination of PFAS. The need for multiple systems to be present simultaneously is shown. First, the fluorinated compound, denoted C–F, may enter the cell passively based on uptake studies. The mechanism of defluorination in this example is reductive. That may require a cofactor such as cobalamin and a low‐potential ferredoxin reducing system. Both the low‐potential electron generating system and the reductive dehalogenase may require ATP. Fluoride, if remaining in the cell, can be inhibitory to the required ATPase, preventing new ATP generation. Fluoride can also inhibit essential cellular enzymes such as pyrophosphatase and enolase. To protect against fluoride toxicity, the cell must sense fluoride, perhaps using a fluoride riboswitch regulatory system, and synthesize a membrane‐bound fluoride exporter system to maintain a low, steady‐state intracellular fluoride concentration.

What are the mechanisms of fluoride toxicity? In mammals, fluoride can induce oxidative stress, disrupt redox homeostasis, increase protein carbonyl content, alter gene expression and cause apoptosis (Barbier *et al*., 2010). In bacteria, cellular disruption is largely attributable to inhibition of essential and non‐essential enzymes (Wiseman, 1970). This has been extensively studied in oral bacteria and is one of the reason for the presence of fluoride in toothpastes (Hamilton, 1990). Fluoride is highly inhibitory to metallo‐enzymes, particularly those using magnesium. The inhibition of specific enzymes can be exceedingly strong, with K_I_ values for some enzymes measured to be as low as 2 μM (Guranowski, 1990). Essential enzymes shown to be inhibited by fluoride include pyrophosphatase (Baykov *et al*., 2000), proton translocating ATPases (Sutton *et al*., 1987; Sturr and Marquis, 1990) and enolase (Qin *et al*., 2006) (Fig. 5). The urease of *Klebsiella aerogenes* has been shown to be inhibited by fluoride (Todd and Hausinger, 2000).

These findings over the last four decades raise an important question regarding the biodegradation of polyfluorinated compounds. Reductive defluorination and other reactions requiring complex enzyme machinery will almost invariably occur intracellularly and release fluoride, so this is another impediment to microbes degrading highly fluorinated compounds. The intracellular volume of a typical bacterial cell is less that 10^−12^ μl (Ingraham *et al*., 1983). Therefore, if there were 10^8^ bacteria per ml and they released one fluorine atom while degrading 10 μM of a fluorinated compound from their surrounding solution, the initial intracellular fluoride concentration would be 100 mM, a highly toxic level. Many PFAS compounds contain ten or more fluorine atoms, compounding the problem.

Fortunately, some bacteria have evolved protective mechanisms of fluoride resistance (Chouhan *et al*., 2012; Liao *et al*., 2015; Mukherjee *et al*., 2017; Chellaiah *et al*., 2021), and these systems would need to be very effective to protect the cell while biodegrading even low μM levels of polyfluorinated compounds. Given that fluorinated natural products and defluorinating enzymes are rare, this protection has likely evolved to protect against external fluoride. There are many regions in the world where fluoride exceeds healthy levels for humans, and bacterial enzymes would be impacted at those same levels (Amini *et al*., 2008). Fluoride uptake in bacteria is impacted by the strong H‐bonding propensity of fluoride in water, which causes it to interact more strongly with protons. That raises the pKa to 3.4, which is much higher than the pKa for other hydrogen halides (O’Hagan, 2008). Under mildly acidic aqueous extracellular conditions, sufficient HF will be present. HF readily partitions into bacterial cells, and free fluoride is released under the more neutral pH of the cytoplasm (Ji *et al*., 2014). If millimolar concentrations are reached, bacterial enzymes will be severely inhibited, as discussed previously.

Two responses are needed to protect the bacterial cells. First, fluoride needs to be sensed. Second, fluoride detection needs to be followed by responses that mitigate against toxic effects. For the latter, the cell is best served by removing fluoride from the cell as a number of enzymes are reversibly inhibited by fluoride, allowing recovery of activity. On the first point, thousands of fluoride‐sensing riboswitches (Fig. 5) have been discovered in many diverse bacterial strains (Baker *et al*., 2012; Breaker, 2012). Riboswitches are components of certain mRNAs that bind to ligands and subsequently regulate protein expression from that mRNA. Proteins expressed via fluoride riboswitches include higher copy numbers of enolase, stress genes, DNA repair functions and hypothetical proteins. Ultimately, the cell can be rescued from fluoride by producing one or both of two non‐homologous classes of transport proteins (Stockbridge *et al*., 2012; Ji *et al*., 2014; Last *et al*., 2018). One such type of transport is a passive, gradient‐controlled passage of fluoride via what is known as Fluc‐F^−^ channels. The other class are F^−^/H^+^ antiporters that act similarly to multi‐drug resistance transporters and use a proton gradient or ATP hydrolysis to expel fluoride against a gradient (Fig. 5).

The toxicity of fluoride raises a key issue for any newly evolving C–F bond cleavage metabolism. Without rapid export of the fluoride product, the metabolism will be counter‐selected against and not be retained in populations. Therefore, this imposes another constraint on biodegradation. In total, effective biodegradation might require a powerful enzyme(s), ATP‐consumption to drive low‐potential C–F bond cleavage and fluoride sensing and export capabilities (Fig. 5). Interestingly, *Acidmicrobium* sp. A6, which biodegrades PFAS compounds (Huang and Jaffé, 2019), has a putative fluoride ion transporter gene(s) in its genome (S. Huang & P. Jaffé, personal communication).

## Prospects and hope

This review has related that polyfluorinated compounds, including PFAS, are biodegradable, the metabolism has not evolved over eons like that for other organohalides, and the chemistry and biology of fluorine make PFAS biodegradation rare. First, microbes have not been long‐exposed to highly‐fluorinated natural products, requiring newly evolved metabolism. Second, evolution and gene spread is driven by natural selection, and metabolism of polyfluorinated compounds may often lack selective benefit. Many PFAS are at or near the carbon dioxide oxidation level, so they are not oxidizable for energy. Moreover, they cannot serve as a final electron acceptor if the redox potential is low enough to preclude a thermodynamic benefit to reduction. Indeed, C–F bond cleavage of multiply fluorinated compounds may require ATP and thus impose a metabolic burden (Fig. 5). If these metabolic impediments hold, then biodegradation will largely be relegated to metabolic ‘accidents.’ That is, high‐powered metabolic enzymes, designed by nature for other metabolism, will potentially cross‐over and react with some polyfluorinated compounds. Third, multiple systems will likely need to be in place for sustained biodegradation. In this context, biodegradation requirements could include low potential redox transfer proteins, enzymes capable of reacting with C–F bonds, transport into the intracellular space and robust fluoride resistance mechanisms (Fig. 5). The latter likely includes active fluoride export proteins and enzymes that are intrinsically more resistant against fluoride inhibition.

Currently, remediation biotechnology uses commercially available cultures of *Dehalococcoides* for organochloride cleanup (Kanitkar *et al*., 2016), and it is important, in concluding this review, to consider if organofluorine compounds might also be microbiologically remediated. The tough questions here focus on PFAS, the most recalcitrant of the organofluorine compounds. For that deliberation, it is necessary to address the following questions: (1) Do PFAS degrading organisms exist? (2) Can the mechanisms of those PFAS degraders be elucidated? (3) Can PFAS degrading bacteria ultimately be harnessed for bioremediation?

For the first point, bacteria have already been demonstrated to biodegrade PFAS (Huang and Jaffé, 2019; Yu *et al*., 2020). Given that there are estimated to be greater than 10^30^ prokaryotes on earth (Whitman *et al*., 1998), the vast majority of which are yet unstudied, there are likely to be more PFAS‐metabolizing bacteria uncovered. For the second point, if microorganisms can be shown to biodegrade PFAS in the laboratory, it is reasonable to expect that the genes and enzymes can be identified and studied in detail. The third question is more difficult to answer. The rates of PFAS degradation reported to date are very low (Huang and Jaffé, 2019; Yu *et al*., 2020), and, currently, they may not be suitable for bioremediation. This impediment could be overcome with discoveries of new organisms or from laboratory engineered microbes derived from insights into the enzymes and mechanisms. There may be questions as to whether regulatory agencies would allow genetically engineered bacteria to be used in PFAS bioremediation, but that is a political, not a scientific, issue. In conclusion, this author is hopeful that real‐world solutions will be forthcoming.

## Conflict of interest

None declared.

## References

[mbt213928-bib-0001] Adamek, E. , Pawłowska‐Góral, K. , and Bober, K. (2005) *In vitro* and *in vivo* effects of fluoride ions on enzyme activity. Ann Acad Med Stetin 51: 69–85. 16519100

[mbt213928-bib-0002] Agarwal, V. , Miles, Z.D. , Winter, J.M. , Eustáquio, A.S. , El Gamal, A.A. , and Moore, B.S. (2017) Enzymatic halogenation and dehalogenation reactions: pervasive and mechanistically diverse. Chem Rev 117: 5619–5674. 28106994 10.1021/acs.chemrev.6b00571 PMC5575885

[mbt213928-bib-0003] Ahrens, L. , and Bundschuh, M. (2014) Fate and effects of poly‐ and perfluoroalkyl substances in the aquatic environment: a review. Environ Toxicol Chem 33: 1921–1929. 24924660 10.1002/etc.2663

[mbt213928-bib-0004] Aigueperse, J. , Mollard, P. , Devilliers, D. , Chemla, M. , Faron, R. , Romano, R.E. , and Cue, J.P. (2000) Fluorine compounds, inorganic. In Ullmann's Encyclopedia of Industrial Chemistry. Weinheim: Wiley‐VCH, pp. 397–441.

[mbt213928-bib-0005] Alexander, M. (1999) Biodegradation and Bioremediation (2nd edn.). Cambridge, MA: Academic Press.

[mbt213928-bib-0006] Alexandrino, D.A.M. , Ribeiro, I. , Pinto, L.M. , Cambra, R. , Oliveira, R.S. , Pereira, F. , and Carvalho, M.F. (2018) Biodegradation of mono‐, di‐ and trifluoroacetate by microbial cultures with different origins. Nat Biotechnol 43: 23–29. 10.1016/j.nbt.2017.08.005 28851570

[mbt213928-bib-0007] Al‐Harthy, T. , Zoghaib, W. , and Abdel‐Jalil, R. (2020) Importance of fluorine in benzazole compounds. Molecules 25: 4677–4696. 10.3390/molecules25204677 PMC7587361 33066333

[mbt213928-bib-0008] Allegre, C.J. , Poirier, J.‐P. , Humler, E. , and Hofmann, A.W. (1995) The chemical composition of the Earth. Earth Planet Sci Lett 134: 515–526.

[mbt213928-bib-0009] Amini, M. , Mueller, K.I.M. , Abbaspour, K.C. , Rosenberg, T. , Afyuni, M. , Møller, K.N. , *et al*. (2008) Statistical modeling of global geogenic fluoride contamination in groundwaters. Environ Sci Technol 42: 3662–3668. 18546705 10.1021/es071958y

[mbt213928-bib-0010] Baker, J.L. , Sudarsan, N. , Weinberg, Z. , Roth, A. , Stockbridge, R.B. , and Breaker, R.R. (2012) Widespread genetic switches and toxicity resistance proteins for fluoride. Science 335: 233–235. 22194412 10.1126/science.1215063 PMC4140402

[mbt213928-bib-0011] Barbier, O. , Arreola‐Mendoza, L. , and Del Razo, L.M. (2010) Molecular mechanisms of fluoride toxicity. Chemico‐Biol Interact 188: 319–333. 10.1016/j.cbi.2010.07.011 20650267

[mbt213928-bib-0012] Batelle (2021) PFAS assessment and mitigation [WWW document]. URL https://www.battelle.org/inb/pfas.

[mbt213928-bib-0013] Baykov, A.A. , Fabrichniy, I.P. , Pohjanjoki, P. , Zyryanov, A.B. , and Lahti, R. (2000) Fluoride effects along the reaction pathway of pyrophosphatase: evidence for a second enzyme⊙ pyrophosphate intermediate. Biochemistry 39: 11939–11947. 11009607 10.1021/bi000627u

[mbt213928-bib-0014] Belkouteb, N. , Franke, V. , McCleaf, P. , Köhler, S. , and Ahrens, L. (2020) Removal of per‐ and polyfluoroalkyl substances (PFASs) in a full‐scale drinking water treatment plant: Long‐term performance of granular activated carbon (GAC) and influence of flow‐rate. Water Res 182: 115913–115923. 32585466 10.1016/j.watres.2020.115913

[mbt213928-bib-0015] Bhatnagar, M. , and Bhatnagar, A. (2004) Physiology of *Anabaena khannae* and *Chlorococcum humicola* under fluoride stress. Folia Microbiol (Praha) 49: 291–296. 15259770 10.1007/BF02931045

[mbt213928-bib-0016] Blackburn, A.C. , Tzeng, H.F. , Anders, M.W. , and Board, P.G. (2000) Discovery of a functional polymorphism in human glutathione transferase zeta by expressed sequence tag database analysis. Pharmacogenetics 10: 49–57. 10739172 10.1097/00008571-200002000-00007

[mbt213928-bib-0017] Bondar, V.S. , Boersma, M.G. , Golovlev, E.L. , Vervoort, J. , Van Berkel, W.J. , Finkelstein, Z.I. , *et al*. (1998) ^19^F NMR study on the biodegradation of fluorophenols by various *Rhodococcus* species. Biodegradation 9: 475–486. 10335585 10.1023/a:1008391906885

[mbt213928-bib-0018] Brase, R.A. , Mullin, E.J. , and Spink, D.C. (2021) Legacy and emerging per‐ and polyfluoroalkyl substances: Analytical techniques, environmental fate, and health effects. Int J Mol Sci 22: 995. 33498193 10.3390/ijms22030995 PMC7863963

[mbt213928-bib-0019] Breaker, R.R. (2012) New insight on the response of bacteria to fluoride. Caries Res 46: 78–81. 22327376 10.1159/000336397 PMC3331882

[mbt213928-bib-0020] Brown, J.F. Jr , Bedard, D.L. , Brennan, M.J. , Carnahan, J.C. , Feng, H. , and Wagner, R.E. (1987) PCB dechlorination in aquatic sediments. Science 236: 709–712. 17748310 10.1126/science.236.4802.709

[mbt213928-bib-0021] Burriss, A. , Edmunds, A.J. , Emery, D. , Hall, R.G. , Jacob, O. , and Schaetzer, J. (2018) The importance of trifluoromethyl pyridines in crop protection. Pest Manag Sci 74: 1228–1238. 29193648 10.1002/ps.4806

[mbt213928-bib-0022] Busto, M.D. , Smith, P.P. , Pérez‐Mateos, M. , and Burns, R.G. (1992) Degradation of aliphatic halogen‐substituted pesticides by dehalogenase isolated from *Pseudomonas alcaligenes*. Identification and properties of the enzyme. Sci Total Environ 123–124: 267–277. 10.1016/0048-9697(92)90152-i 1439734

[mbt213928-bib-0023] Camargo, J.A. (2003) Fluoride toxicity to aquatic organisms: a review. Chemosphere 50: 251–264. 12656244 10.1016/s0045-6535(02)00498-8

[mbt213928-bib-0237] Castro, C.E. , and Belser, N.O. (1968) Biodehalogenation. Reductive dehalogenation of the biocides ethylene dibromide, 1,2‐dibromo‐3‐chloropropane, and 2,3‐dibromobutane in soil. Environ Sci Technol 2: 779–783. 26505514 10.1021/es60173a019

[mbt213928-bib-0024] Chan, P.W. , Yakunin, A.F. , Edwards, E.A. , and Pai, E.F. (2011) Mapping the reaction coordinates of enzymatic defluorination. J Am Chem Soc 133: 7461–7468. 21510690 10.1021/ja200277d PMC3101105

[mbt213928-bib-0025] Che, S. , Jin, B. , Liu, Z. , Yu, Y. , Liu, J. , and Men, Y. (2021) Structure‐specific aerobic defluorination of short‐chain fluorinated carboxylic acids by activated sludge communities. Environ Sci Technol Lett 8: 668–674. 10.1021/acs.estlett.1c00511 PMC8936751 35316934

[mbt213928-bib-0026] Chellaiah, E.R. , Ravi, P. , and Uthandakalaipandian, R. (2021) High fluoride resistance and virulence profile of environmental *Pseudomonas* isolated from water sources. Folia Microbiol (Praha) 66: 1–10. 33821405 10.1007/s12223-021-00867-z

[mbt213928-bib-0027] Chen, G. , Jiang, N. , Villalobos‐Solis, M.I. , Kara‐Murdoch, F. , Murdoch, R.W. , Xie, Y. , *et al*. (2021) Anaerobic microbial metabolism of dichloroacetate. MBio 12: e00537‐21. 33906923 10.1128/mBio.00537-21 PMC8092247

[mbt213928-bib-0028] Chen, T.Y. (2005) Structure and function of clc channels. Annu Rev Physiol 67: 809–839. 15709979 10.1146/annurev.physiol.67.032003.153012

[mbt213928-bib-0029] Chesler, R.D. , Clarke, A.J. , and Kelliher, R. (2021) PFAS: liability and insurance coverage issues for the “FOREVER CHEMICAL”. Environ Claims J 33: 136–148.

[mbt213928-bib-0030] Ching, C. , Klemes, M.J. , Trang, B. , Dichtel, W.R. , and Helbling, D.E. (2020) β‐Cyclodextrin polymers with different crosslinkers and ion exchange resins exhibit variable adsorption of anionic, zwitterionic, and nonionic PFASs. Environ Sci Technol 54: 12693–12702. 32924449 10.1021/acs.est.0c04028

[mbt213928-bib-0031] Chouhan, S. , Tuteja, U. , and Flora, S.J.S. (2012) Isolation, identification and characterization of fluoride resistant bacteria: possible role in bioremediation. Appl Biochem Microbiol 48: 43–50. 22567885

[mbt213928-bib-0032] Clark, J. (2002) The acidity of the hydrogen halides. chemguide.co.uk. Retrieved July 1, 2021.

[mbt213928-bib-0033] Copley, S.D. (2009) Evolution of efficient pathways for degradation of anthropogenic chemicals. Nat Chem Biol 5: 559–566. 19620997 10.1038/nchembio.197 PMC2867350

[mbt213928-bib-0034] Cordner, A. , Goldenman, G. , Birnbaum, L.S. , Brown, P. , Miller, M.F. , Mueller, R. , *et al*. (2021) The true cost of PFAS and the benefits of acting now. Environ Sci Technol 55: 9630–9633. 34231362 10.1021/acs.est.1c03565 PMC8296683

[mbt213928-bib-0035] Dalvi, V.H. , and Rossky, P.J. (2010) Molecular origins of fluorocarbon hydrophobicity. Proc Natl Acad Sci USA 107: 13603–13607. 20643968 10.1073/pnas.0915169107 PMC2922239

[mbt213928-bib-0036] Davis, C.K. , Webb, R.I. , Sly, L.I. , Denman, S.E. , and McSweeney, C.S. (2012) Isolation and survey of novel fluoroacetate‐degrading bacteria belonging to the phylum Synergistetes. FEMS Microbiol Ecol 80: 671–684. 22372434 10.1111/j.1574-6941.2012.01338.x

[mbt213928-bib-0037] Dawson, C.D. , Irwin, S.M. , Backman, L.R.F. , Le, C. , Wang, J.X. , Vennelakanti, V. , *et al*. (2021) Molecular basis of C‐S bond cleavage in the glycyl radical enzyme isethionate sulfite‐lyase. Cell Chem Biol 19: S2451–9456. 10.1016/j.chembiol.2021.03.001 PMC8473560 33773110

[mbt213928-bib-0039] Dinglasan, M.J.A. , Ye, Y. , Edwards, E.A. , and Mabury, S.A. (2004) Fluorotelomer alcohol biodegradation yields poly‐and perfluorinated acids. Environ Sci Technol 38: 2857–2864. 15212260 10.1021/es0350177

[mbt213928-bib-0040] Dionizio, A. , Uyghurturk, D.A. , Melo, C.G.S. , Sabino‐Arias, I.T. , Araujo, T.T. , Ventura, T.M.S. , *et al*. (2021) Intestinal changes associated with fluoride exposure in rats: integrative morphological, proteomic and microbiome analyses. Chemosphere 273: 129607. 33508686 10.1016/j.chemosphere.2021.129607 PMC8076095

[mbt213928-bib-0041] Dixon, D.P. , Cole, D.J. , and Edwards, R. (2000) Characterisation of a zeta class glutathione transferase from *Arabidopsis thaliana* with a putative role in tyrosine catabolism. Arch Biochem Biophys 384: 407–412. 11368331 10.1006/abbi.2000.2125

[mbt213928-bib-0042] Dobbs, A.P. , and Kimberley, M.R. (2002) Fluorous phase chemistry: a new industrial technology. J Fluorine Chem 118: 3–17.

[mbt213928-bib-0043] Dolbier, W.R. (2005) Fluorine chemistry at the millenium. J Fluorine Chem 126: 157–163.

[mbt213928-bib-0044] Dong, H. , Dong, S. , Erik‐Hansen, P. , Stagos, D. , Lin, X. , and Liu, M. (2020) Progress of bromophenols in marine algae from 2011 to 2020: structure, bioactivities, and applications. Mar Drugs 18: 411. 10.3390/md18080411 PMC7459620 32759739

[mbt213928-bib-0045] Donnelly, C. , and Murphy, C.D. (2009) Purification and properties of fluoroacetate dehalogenase from *Pseudomonas fluorescens* DSM 8341. Biotechnol Lett 31: 245–250. 18807226 10.1007/s10529-008-9849-4

[mbt213928-bib-0046] Duhamel, M. , and Edwards, E.A. (2007) Growth and yields of dechlorinators, acetogens, and methanogens during reductive dechlorination of chlorinated ethenes and dihaloelimination of 1, 2‐dichloroethane. Environ Sci 41: 2303–2310. 10.1021/es062010r 17438779

[mbt213928-bib-0047] Dvořák, P. , Nikel, P.I. , Damborský, J. , and de Lorenzo, V. (2017) Bioremediation 3.0: engineering pollutant‐removing bacteria in the times of systemic biology. Biotech Adv 35: 845–866. 10.1016/j.biotechadv.2017.08.001 28789939

[mbt213928-bib-0048] Edelbach, B.L. , and Jones, W.D. (1997) Mechanism of carbon−fluorine bond activation by (C5Me5) Rh (PMe3) H2. J Am Chem Soc 119: 7734–7742.

[mbt213928-bib-0049] Edmunds, W.M. , and Smedley, P.L. (2013) Fluoride in natural waters. In Essentials of Medical Geology. Dordrecht, The Netherlands: Springer, pp. 311–336.

[mbt213928-bib-0050] Eisenstein, O. , Milani, J. , and Perutz, R.N. (2017) Selectivity of C‐H activation and competition between C–H and C–F bond activation at fluorocarbons. Chem Rev 117: 8710–8753. 28653537 10.1021/acs.chemrev.7b00163

[mbt213928-bib-0051] Emsley, J. (2011) Nature's Building Blocks: An A‐Z Guide to the Elements (2nd edn). Oxford: Oxford University Press.

[mbt213928-bib-0052] Engesser, K.H. , and Schulte, P. (1989) Degradation of 2‐bromo‐, 2‐chloro‐and 2‐fluorobenzoate by *Pseudomonas putida* CLB 250. FEMS Microbiol Lett 60: 143–147. 10.1016/0378-1097(89)90497-7 2777062

[mbt213928-bib-0053] Envirogen Technologies (2021) Per‐ and polyfluoroalkyl substances (PFAS) contamination – water treatment [WWW document] URL https://www.envirogen.com/contaminants/per‐and‐polyfluoroalkyl‐substances‐pfas/

[mbt213928-bib-0054] Fedorenko, M. , Alesio, J. , Fedorenko, A. , Slitt, A. , and Bothun, G.D. (2021) Dominant entropic binding of perfluoroalkyl substances (PFASs) to albumin protein revealed by ^19^F NMR. Chemosphere 263: 128083. 33297081 10.1016/j.chemosphere.2020.128083 PMC8479757

[mbt213928-bib-0055] Fejzagić, A.V. , Gebauer, J. , Huwa, N. , and Classen, T. (2019) Halogenating enzymes for active agent synthesis: first steps are done and many have to follow. Molecules 24: 4008. 10.3390/molecules24214008 PMC6864650 31694313

[mbt213928-bib-0056] Filho, A.H.D.S. , and de Souza, G.L.C. (2020) Examining the degradation of environmentally daunting per‐ and poly‐fluoroalkyl substances from a fundamental chemical perspective. Phys Chem Chem Phys 22: 17659–17667. 32724980 10.1039/d0cp02445g

[mbt213928-bib-0057] Fincker, M. , and Spormann, A.M. (2017) Biochemistry of catabolic reductive dehalogenation. Ann Rev Biochem 86: 357–386. 28654328 10.1146/annurev-biochem-061516-044829

[mbt213928-bib-0058] Fitzgerald, N.J. , Simcik, M.F. , and Novak, P.J. (2018) Perfluoroalkyl substances increase the membrane permeability and quorum sensing response in *Aliivibrio fischeri* . Environ Sci Technol Lett 5: 26–31.

[mbt213928-bib-0059] Fitzgerald, N.J. , Wargenau, A. , Sorenson, C. , Pedersen, J. , Tufenkji, N. , Novak, P.J. , and Simcik, M.F. (2018) Partitioning and accumulation of perfluoroalkyl substances in model lipid bilayers and bacteria. Environ Sci Technol 52: 10433–10440. 30148610 10.1021/acs.est.8b02912

[mbt213928-bib-0060] Fox, B. , Borneman, J.G. , Wackett, L.P. , and Lipscomb, J.D. (1990) Haloalkene oxidation by the soluble methane monooxygenase from *Methylosinus trichosporium* OB3b: Mechanistic and environmental implications. Biochemistry 29: 6419–6427. 2207083 10.1021/bi00479a013

[mbt213928-bib-0061] Futagami, T. , Goto, M. , and Furukawa, K. (2008) Biochemical and genetic bases of dehalorespiration. Chem Rec 8: 1–12. 18302277 10.1002/tcr.20134

[mbt213928-bib-0062] Gälli, R. , and McCarty, P.L. (1989) Biotransformation of 1,1,1‐trichloroethane, trichloromethane, and tetrachloromethane by a *Clostridium* sp. Appl Environ Microbiol 55: 837–844. 2729985 10.1128/aem.55.4.837-844.1989 PMC184211

[mbt213928-bib-0063] Gao, X.T. , Zhang, Z. , Wang, X. , Tian, J.S. , Xie, S.L. , Zhou, F. , and Zhou, J. (2020) Direct electrochemical defluorinative carboxylation of α‐CF 3 alkenes with carbon dioxide. Chem Sci 11: 10414–10420. 34123181 10.1039/d0sc04091f PMC8162267

[mbt213928-bib-0064] Gardella, J. (2020) Are the floodgates open for PFAS product liability cases? Natl Law Rev XI (94). https://www.natlawreview.com/article/pfas‐product‐liability‐cases‐are‐floodgates‐now‐open.

[mbt213928-bib-0065] Glüge, J. , Scheringer, M. , Cousins, I.T. , DeWitt, J.C. , Goldenman, G. , Herzke, D. , *et al*. (2020) An overview of the uses of per‐and polyfluoroalkyl substances (PFAS). Environ Sci Process Impact 22: 2345–2373. 10.1039/d0em00291g PMC7784712 33125022

[mbt213928-bib-0066] Goldman, P. (1965) The enzymatic cleavage of the carbon‐fluorine bond in fluoroacetate. J Biol Chem 240: 3434–3438. 14321384

[mbt213928-bib-0067] Grandjean, P. , Timmermann, C.A.G. , Kruse, M. , Nielsen, F. , Vinholt, P.J. , Boding, L. , *et al*. (2020) Severity of COVID‐19 at elevated exposure to perfluorinated alkylates. PLoS One 15: e0244815. 33382826 10.1371/journal.pone.0244815 PMC7774856

[mbt213928-bib-0068] Greenhalgh, R. , and Riley, J.P. (1961) The determination of fluorides in natural waters, with particular reference to sea water. Anal Chim Acta 25: 179–188.

[mbt213928-bib-0069] Gribble, G.W. (2012) Occurrence of halogenated alkaloids. Alkaloids Chem Biol 71: 1–165. 23189746 10.1016/b978-0-12-398282-7.00001-1

[mbt213928-bib-0070] Gribble, G.W. (2015) Biological activity of recently discovered halogenated marine natural products. Mar Drugs 13: 4044. 26133553 10.3390/md13074044 PMC4515607

[mbt213928-bib-0071] Gulick, A.M. , Hubbard, B.K. , Gerlt, J.A. , and Rayment, I. (2001) Evolution of enzymatic activities in the enolase ruperfamily: identification of the general acid catalyst in the actives of D‐glucarate dehydratase from *Escherichia coli* . Biochemistry 40: 10054–10062. 11513584 10.1021/bi010733b

[mbt213928-bib-0072] Guranowski, A. (1990) Fluoride is a strong and specific inhibitor of (asymmetrical) Ap4A hydrolases. FEBS Lett 262: 205–208. 2159411 10.1016/0014-5793(90)80190-t

[mbt213928-bib-0073] Hagmann, W.K. (2008) The many roles for fluorine in medicinal chemistry. J Med Chem 51: 4359–4369. 18570365 10.1021/jm800219f

[mbt213928-bib-0074] Hamilton, I.R. (1990) Biochemical effects of fluoride on oral bacteria. J Dental Res 69: 660–667. 10.1177/00220345900690S128 2179327

[mbt213928-bib-0238] Harkey, A. , Kim, H. , Kandagatla, S. , and Raner, G.M. (2012) Defluorination of 4‐fluorophenol by cytochrome P450 BM3‐F87G: activation by long chain fatty aldehydes. Biotech Lett 34: 1725–1731. 10.1007/s10529-012-0957-9 22639088

[mbt213928-bib-0075] Hasan, S.A. , Ferreira, M.I. , Koetsier, M.J. , Arif, M.I. , and Janssen, D.B. (2011) Complete biodegradation of 4‐fluorocinnamic acid by a consortium comprising *Arthrobacter* sp. strain G1 and *Ralstonia* sp. strain H1. Appl Environ Microbiol 77: 572–579. 21097599 10.1128/AEM.00393-10 PMC3020533

[mbt213928-bib-0076] Hevey, R. (2021) The role of fluorine in glycomimetic drug design. Chemistry 27: 2240–2253. 32901973 10.1002/chem.202003135

[mbt213928-bib-0077] Hogue, C. (2021) PFAS targeted in legislation passed by US House of Representatives. Chem Eng News: 6–7. July 21, 2021.

[mbt213928-bib-0078] Holliger, C. , Wohlfarth, G. , and Diekert, G. (1998) Reductive dechlorination in the energy metabolism of anaerobic bacteria. FEMS Microbiol Rev 22: 383–398.

[mbt213928-bib-0079] Hou, R. , Lin, L. , Li, H. , Liu, S. , Xu, X. , Xu, Y. , *et al*. (2021) Occurrence, bioaccumulation, fate, and risk assessment of novel brominated flame retardants (NBFRs) in aquatic environments—a critical review. Water Res 198: 117168. 33962238 10.1016/j.watres.2021.117168

[mbt213928-bib-0080] Huang, S. , and Jaffé, P.R. (2019) Defluorination of perfluorooctanoic acid (PFOA) and perfluorooctane sulfonate (PFOS) by *Acidimicrobium* sp. strain A6. Environ Sci Technol 53: 11410–11419. 31529965 10.1021/acs.est.9b04047

[mbt213928-bib-0081] Hug, L.A. , Maphosa, F. , Leys, D. , Löffler, F.E. , Smidt, H. , Edwards, E.A. , and Adrian, L. (2013) Overview of organohalide‐respiring bacteria and a proposal for a classification system for reductive dehalogenases. Philos Trans R Soc Lond B Biol Sci 368: 20120322. 23479752 10.1098/rstb.2012.0322 PMC3638463

[mbt213928-bib-0082] Hügel, H.M. , and Jackson, N. (2012) Special feature: organo‐fluorine chemical science. Appl Sci 2: 558–565.

[mbt213928-bib-0083] Huwiler, S.G. , Löffler, C. , Anselmann, S.E. , Stärk, H.J. , von Bergen, M. , Flechsler, J. , *et al*. (2019) One‐megadalton metalloenzyme complex in *Geobacter metallireducens* involved in benzene ring reduction beyond the biological redox window. Proc Natl Acad Sci USA 116: 2259–2264. 30674680 10.1073/pnas.1819636116 PMC6369795

[mbt213928-bib-0084] Ingraham, J.L. , Maaløe, O. , and Neidhardt, F.C. (1983) Growth of the bacterial cell. Sinauer Associates.

[mbt213928-bib-0085] Inoue, K. , Kato, Y. , and Kandori, H. (2015) Light‐driven ion‐translocating rhodopsins in marine bacteria. Trends Microbiol 23: 91–98. 25432080 10.1016/j.tim.2014.10.009

[mbt213928-bib-0086] Iyer, R. , Iverson, T.M. , Accardi, A. , and Miller, C. (2002) A biological role for prokaryotic ClC chloride channels. Nature 419: 715–718. 12384697 10.1038/nature01000

[mbt213928-bib-0087] Jeschke, P. (2004) The unique role of fluorine in the design of active ingredients for modern crop protection. ChemBioChem 5: 570–589. 10.1002/cbic.200300833 15122630

[mbt213928-bib-0088] Jeschke, P. (2017) Latest generation of halogen‐containing pesticides. Pest Manag Sci 73: 1053–1066. 28145087 10.1002/ps.4540

[mbt213928-bib-0089] Ji, C. , Stockbridge, R.B. , and Miller, C. (2014) Bacterial fluoride resistance, Fluc channels and the weak acid accumulation effect. J Gen Physiol 144: 257. 25156118 10.1085/jgp.201411243 PMC4144673

[mbt213928-bib-0090] Johnston, N.R. , and Strobel, S.A. (2020) Principles of fluoride toxicity and the cellular response: a review. Arch Toxicol 94: 1051–1069. 32152649 10.1007/s00204-020-02687-5 PMC7230026

[mbt213928-bib-0091] Jugder, B.E. , Ertan, H. , Bohl, S. , Lee, M. , Marquis, C.P. , and Manefield, M. (2016) Organohalide respiring bacteria and reductive dehalogenases: key tools in organohalide bioremediation. Front Microbiol 7: 249. 26973626 10.3389/fmicb.2016.00249 PMC4771760

[mbt213928-bib-0092] Kanitkar, Y.H. , Stedtfeld, R.D. , Steffan, R.J. , Hashsham, S.A. , and Cupples, A.M. (2016) Loop‐mediated isothermal amplification (LAMP) for rapid detection and quantification of *Dehalococcoides* biomarker genes in commercial reductive dechlorinating cultures KB‐1 and SDC‐9. Appl Environ Microbiol 82: 1799–1806. 26746711 10.1128/AEM.03660-15 PMC4784023

[mbt213928-bib-0093] Key, B.D. , Howell, R.D. , and Criddle, C.S. (1997) Fluorinated organics in the biosphere. Environ Sci Technol 31: 2445–2454.

[mbt213928-bib-0236] Kiel, M. , and Engesser, K.H. (2015) The biodegradation vs. biotransformation of fluorosubstituted aromatics. Appl Microbiol Biotech 99: 7433–7464. 10.1007/s00253-015-6817-5 26216240

[mbt213928-bib-0094] Kim, S. , Chen, J. , Cheng, T. , Gindulyte, A. , He, J. , He, S. , *et al*. (2021) PubChem in 2021: new data content and improved web interfaces. Nucleic Acids Res 49: D1388–D1395. 33151290 10.1093/nar/gkaa971 PMC7778930

[mbt213928-bib-0095] Kiplinger, J.L. , Richmond, T.G. , and Osterberg, C.E. (1994) Activation of carbon‐fluorine bonds by metal complexes. Chem Rev 94: 373–431.

[mbt213928-bib-0096] Kirsch, P. (2004) Modern Fluoroorganic Chemistry: Synthesis, Reactivity, Applications. Weinheim, Germany: Wiley‐VCH.

[mbt213928-bib-0097] Kissa, E. (2001) Fluorinated Surfactants and Repellents (vol. 97). Boca Raton, FL: CRC Press.

[mbt213928-bib-0098] Kräutler, B. , Fieber, W. , Ostermann, S. , Fasching, M. , Ongania, K.H. , Gruber, K. , *et al*. (2003) The cofactor of tetrachloroethene reductive dehalogenase of *Dehalospirillum multivorans* is norpseudo‐B12, a new type of a natural corrinoid. Helv Chim Acta 86: 3698–3716.

[mbt213928-bib-0099] Küpper, F.C. , and Carrano, C.J. (2019) Key aspects of the iodine metabolism in brown algae: a brief critical review. Metallomics 11: 756–764. 30834917 10.1039/c8mt00327k

[mbt213928-bib-0100] Kurihara, T. , Esaki, N. , and Soda, K. (2000) Bacterial 2‐haloacid dehalogenases: structures and reaction mechanisms. J Mol Catal B Enzymatic 10: 57–65.

[mbt213928-bib-0101] Kurihara, T. , Yamauchi, T. , Ichiyama, S. , Takahata, H. , and Esaki, N. (2003) Purification, characterization, and gene cloning of a novel fluoroacetate dehalogenase from *Burkholderia* sp. FA1. J Mol Catal B: Enzymatic 23: 347–355.

[mbt213928-bib-0102] Kwiatkowski, C.F. , Andrews, D.Q. , Birnbaum, L.S. , Bruton, T.A. , DeWitt, J.C. , Knappe, D.R.U. , *et al*. (2020) Scientific basis for managing PFAS as a chemical class. Environ Sci Technol Lett 7: 532–543. 34307722 10.1021/acs.estlett.0c00255 PMC8297807

[mbt213928-bib-0103] Last, N.B. , Stockbridge, R.B. , Wilson, A.E. , Shane, T. , Kolmakova‐Partensky, L. , Koide, A. , *et al*. (2018) A CLC‐type F^‐^/H^+^ antiporter in ion‐swapped conformations. Nat Struct Mol Biol 25: 601. 29941917 10.1038/s41594-018-0082-0 PMC6044475

[mbt213928-bib-0104] Leisinger, T. , Bader, R. , Hermann, R. , Schmid‐Appert, M. , and Vuilleumier, S. (1994) Microbes, enzymes and genes involved in dichloromethane utilization. Biodegradation 5: 237–248. 7765835 10.1007/BF00696462

[mbt213928-bib-0105] Li, G.L. , Nishiguchi, H. , Ishihara, T. , Moro‐Oka, Y. , and Takita, Y. (1998) Catalytic dehydrofluorination of CF_3_CH_3_ (HFC143a) into CF_2_CH_2_ (HFC1132a). Appl Catal B: Environ 16: 309–317.

[mbt213928-bib-0106] Li, J. , Davis, I. , Griffith, W.P. , and Liu, A. (2020) Formation of monofluorinated radical cofactor in galactose oxidase through copper‐mediated C‐F bond scission. J Am Chem Soc 142: 18753–18757. 33091303 10.1021/jacs.0c08992 PMC7737484

[mbt213928-bib-0108] Li, S. , and Wackett, L.P. (1993) Reductive dehalogenation by cytochrome P450CAM: substrate binding and catalysis. Biochemistry 32: 9355–9361. 8369306 10.1021/bi00087a014

[mbt213928-bib-0109] Li, Y. , Yue, Y. , Zhang, H. , Yang, Z. , Wang, H. , Tian, S. , *et al*. (2019) Harnessing fluoroacetate dehalogenase for defluorination of fluorocarboxylic acids: in silico and in vitro approach. Environ Int 131: 104999. 31319293 10.1016/j.envint.2019.104999

[mbt213928-bib-0110] Liang, B. , Jiang, J. , Zhang, J. , Zhao, Y. , and Li, S. (2012) Horizontal transfer of dehalogenase genes involved in the catalysis of chlorinated compounds: evidence and ecological role. Crit Rev Microbiol 38: 95–110. 21967404 10.3109/1040841X.2011.618114

[mbt213928-bib-0111] Liao, Y. , Brandt, B.W. , Li, J. , Crielaard, W. , Van Loveren, C. , and Deng, D.M. (2017) Fluoride resistance in *Streptococcus mutans*: a mini review. J Oral Microbiol 9: 1344509. 28748043 10.1080/20002297.2017.1344509 PMC5508371

[mbt213928-bib-0112] Liao, Y. , Chen, J. , Brandt, B.W. , Zhu, Y. , Li, J. , van Loveren, C. , and Deng, D.M. (2015) Identification and functional analysis of genome mutations in a fluoride‐resistant *Streptococcus mutans* strain. PLoS One 10: e0122630. 25856576 10.1371/journal.pone.0122630 PMC4391945

[mbt213928-bib-0113] Lim, X. (2021) Can microbes save us from PFAS? Chem Eng News: 30–34. 10.1021/acscentsci.1c00013 PMC7844844 33532561

[mbt213928-bib-0114] Liu, C. , Hatton, J. , Arnold, W.A. , Simcik, M.F. , and Pennell, K.D. (2020) *In situ* sequestration of perfluoroalkyl substances using polymer‐stabilized powdered activated carbon. Environ Sci Technol 54: 6929–6936. 32379438 10.1021/acs.est.0c00155

[mbt213928-bib-0115] Liu, D. , Wei, Y. , Liu, X. , Zhou, Y. , Jiang, L. , Yin, J. , *et al*. (2018) Indoleacetate decarboxylase is a glycyl radical enzyme catalysing the formation of malodorant skatole. Nat Commun 9: 4224. 30310076 10.1038/s41467-018-06627-x PMC6181972

[mbt213928-bib-0116] Liu, J. , Van Hoomissen, D.J. , Liu, T. , Maizel, A. , Huo, X. , Fernández, S.R. , *et al*. (2018) Reductive defluorination of branched per‐and polyfluoroalkyl substances with cobalt complex catalysts. Environ Sci Technol Lett 5: 289–294.

[mbt213928-bib-0118] Liu, X. , Tian, J. , Liu, L. , Zhu, T. , Yu, X. , Chu, X. , *et al*. (2017) Identification of an operon involved in fluoride resistance in *Enterobacter cloacae* FRM. Sci Rep 7: 6786. 28754999 10.1038/s41598-017-06988-1 PMC5533749

[mbt213928-bib-0119] Liu, Z. , Bentel, M.J. , Yu, Y. , Ren, C. , Gao, J. , Pulikkal, V.F. , *et al*. (2021) Near‐quantitative defluorination of perfluorinated and fluorotelomer carboxylates and sulfonates with integrated oxidation and reduction. Environ Sci Technol 55: 7052–7062. 33950686 10.1021/acs.est.1c00353

[mbt213928-bib-0120] Löffler, C. , Kuntze, K. , Vazquez, J.R. , Rugor, A. , Kung, J.W. , Böttcher, A. , and Boll, M. (2011) Occurrence, genes and expression of the W/Se‐containing class II benzoyl‐coenzyme A reductases in anaerobic bacteria. Environ Microbiol 13: 696–709. 21087381 10.1111/j.1462-2920.2010.02374.x

[mbt213928-bib-0121] Loh, Z.H. , Ouwerkerk, D. , Klieve, A.V. , Hungerford, N.L. , and Fletcher, M.T. (2020) Toxin degradation by rumen microorganisms: a review. Toxins 12: 664. 10.3390/toxins12100664 PMC7590051 33092236

[mbt213928-bib-0122] Ludewig, H. , Molyneux, S. , Ferrinho, S. , Guo, K. , Lynch, R. , Gkotsi, D.S. , and Goss, R.J. (2020) Halogenases: structures and functions. Curr Opin Struct Biol 65: 51–60. 32619660 10.1016/j.sbi.2020.05.012

[mbt213928-bib-0123] Maduke, M. , Pheasant, D.J. , and Miller, C. (1999) High‐level expression, functional reconstitution, and quarternary structure of a prokaryotic ClC‐type chloride channel. J Gen Physiol 114: 713–722. 10539975 10.1085/jgp.114.5.713 PMC2230540

[mbt213928-bib-0124] Maga, D. , Aryan, V. , and Bruzzano, S. (2021) Environmental assessment of various end‐of‐life pathways for treating per‐ and polyfluoroalkyl substances in spent fire‐extinguishing waters. Environ Toxicol Chem 40: 947–957. 32539177 10.1002/etc.4803

[mbt213928-bib-0125] Manna, R.N. , Zinovjev, K. , Tunon, I. , and Dybala‐Defratyka, A. (2015) Dehydrochlorination of hexachlorocyclohexanes catalyzed by the LinA dehydrohalogenase. A QM/MM study. J Phys Chem B 119: 15100–15109. 26561208 10.1021/acs.jpcb.5b07538

[mbt213928-bib-0126] Mastropietro, T. , Bruno, R. , Pardo, E. , and Armentano, D. (2021) Reverse osmosis and nanofiltration membranes for highly efficient PFASs removal: overview, challenges and future perspectives. Dalton Trans 50: 5398–5410. 33908956 10.1039/d1dt00360g

[mbt213928-bib-0127] Mayer‐Blackwell, K. , Sewell, H. , Fincker, M. , and Spormann, A.M. (2016) Comparative physiology of organohalide‐respiring bacteria. In Organohalide‐respiring Bacteria. Adrian, L. , and Löffler, F.E. (eds). Berlin, Heidelberg: Springer, pp. 259–280.

[mbt213928-bib-0128] McDonough . (2021) The composition of the earth. [WWW document]. URL http://quake.mit.edu/hilstgroup/CoreMantle/EarthCompo.pdf.

[mbt213928-bib-0129] Meanwell, N.A. (2018) Fluorine and fluorinated motifs in the design and application of bioisosteres for drug design. J Med Chem 61: 5822–5880. 29400967 10.1021/acs.jmedchem.7b01788

[mbt213928-bib-0130] Michlits, H. , Lier, B. , Pfanzagl, V. , Djinović‐Carugo, K. , Furtmüller, P.G. , Oostenbrink, C. , *et al*. (2020) Actinobacterial coproheme decarboxylases use histidine as a distal base to promote compound I formation. ACS Catal 10: 5405–5418. 32440366 10.1021/acscatal.0c00411 PMC7235987

[mbt213928-bib-0131] Milton, R.D. , and Minteer, S.D. (2019) Nitrogenase bioelectrochemistry for synthesis applications. Accounts Chem Res 52: 3351–3360. 10.1021/acs.accounts.9b00494 31800207

[mbt213928-bib-0132] Miranda Rojas, S. , Fernández, I. , Kaestner, J. , Labbé, A.T. , and Emaldía, F.M. (2018) Unraveling the nature of the catalytic power of fluoroacetate dehalogenase. ChemCatChem 10: 1052–1063.

[mbt213928-bib-0133] Mohn, W.W. , and Tiedje, J.M. (1990) Strain DCB‐1 conserves energy for growth from reductive dechlorination coupled to formate oxidation. Arch Microbiol 153: 267–271. 2334249 10.1007/BF00249080

[mbt213928-bib-0134] Mondal, D. , Fisher, B.F. , Jiang, Y. , and Lewis, J.C. (2021) Flavin‐dependent halogenases catalyze enantioselective olefin halocyclization. Nat Commun 12: 3268. 34075034 10.1038/s41467-021-23503-3 PMC8169660

[mbt213928-bib-0135] Mukherjee, S. , Yadav, V. , Mondal, M. , Banerjee, S. , and Halder, G. (2017) Characterization of a fluoride‐resistant bacterium *Acinetobacter* sp. RH5 towards assessment of its water defluoridation capability. Appl Water Sci 7: 1923–1930.

[mbt213928-bib-0136] Müller, V. , and Oren, A. (2003) Metabolism of chloride in halophilic prokaryotes. Extremophiles 7: 261–266. 12728360 10.1007/s00792-003-0332-9

[mbt213928-bib-0137] Murphy, C.D. (2010) Biodegradation and biotransformation of organofluorine compounds. Biotech Lett 32: 351–359. 10.1007/s10529-009-0174-3 19943179

[mbt213928-bib-0138] Murphy, C.D. , Schaffrath, C. , and O'Hagan, D. (2003) Fluorinated natural products: the biosynthesis of fluoroacetate and 4‐fluorothreonine in *Streptomyces cattleya* . Chemosphere 52: 455–461. 12738270 10.1016/S0045-6535(03)00191-7

[mbt213928-bib-0139] Nagata, Y. , Mori, K. , Takagi, M. , Murzin, A.G. , and Damborský, J. (2001) Identification of protein fold and catalytic residues of γ‐hexachlorocyclohexane dehydrochlorinase LinA. Proteins Struct Funct Bioinf 45: 471–477. 10.1002/prot.10007 11746694

[mbt213928-bib-0140] Nakayama, T. , Kamachi, T. , Jitsumori, K. , Omi, R. , Hirotsu, K. , Esaki, N. , *et al*. (2012) Substrate specificity of fluoroacetate dehalogenase: an insight from crystallographic analysis, fluorescence spectroscopy, and theoretical computations. Chem Eur J 18: 8392–8402. 22674735 10.1002/chem.201103369

[mbt213928-bib-0141] Nelson, J.W. , Plummer, M.S. , Blount, K.F. , Ames, T.D. , and Breaker, R.R. (2015) Small molecule fluoride toxicity agonists. Chem Biol 22: 527–534. 25910244 10.1016/j.chembiol.2015.03.016 PMC4617673

[mbt213928-bib-0143] Neumann, A. , Wohlfarth, G. , and Diekert, G. (1996) Purification and characterization of tetrachloroethene reductive dehalogenase from *Dehalospirillum multivorans* . J Biol Chem 271: 16515–16519. 8663199 10.1074/jbc.271.28.16515

[mbt213928-bib-0144] Niu, J. , Li, Y. , Shang, E. , Xu, Z. , and Liu, J. (2016) Electrochemical oxidation of perfluorinated compounds in water. Chemosphere 146: 526–538. 26745381 10.1016/j.chemosphere.2015.11.115

[mbt213928-bib-0145] Ochoa‐Herrera, V. , Banihani, Q. , León, G. , Khatri, C. , Field, J.A. , and Sierra‐Alvarez, R. (2009) Toxicity of fluoride to microorganisms in biological wastewater treatment systems. Water Res 43: 3177–3186. 19457531 10.1016/j.watres.2009.04.032

[mbt213928-bib-0146] Ochoa‐Herrera, V. , Field, J.A. , Luna‐Velasco, A. , and Sierra‐Alvarez, R. (2016) Microbial toxicity and biodegradability of perfluorooctane sulfonate (PFOS) and shorter chain perfluoroalkyl and polyfluoroalkyl substances (PFASs). Environ Sci Process Impacts 18: 1236–1246. 27711852 10.1039/c6em00366d

[mbt213928-bib-0147] Ogawa, Y. , Tokunaga, E. , Kobayashi, O. , Hirai, K. , and Shibata, N. (2020) Current contributions of organofluorine compounds to the agrochemical industry. iScience 23: 101467. 32891056 10.1016/j.isci.2020.101467 PMC7479632

[mbt213928-bib-0148] O'Hagan, D. (2008) Understanding organofluorine chemistry. An introduction to the C‐F bond. Chem Soc Rev 37: 308–319. 18197347 10.1039/b711844a

[mbt213928-bib-0149] O'Hagan, D. , and Rzepa, H.S. (1997) Some influences of fluorine in bioorganic chemistry. Chemical Comm 7: 645–652.

[mbt213928-bib-0150] Palma, D. , Papagiannaki, D. , Lai, M. , Binetti, R. , Sleiman, M. , Minella, M. , and Richard, C. (2021) PFAS degradation in ultrapure and groundwater using non‐thermal plasma. Molecules 26: 924. 33572434 10.3390/molecules26040924 PMC7916234

[mbt213928-bib-0151] Pandey, T. , Shukla, R. , Shukla, H. , Sonkar, A. , Tripathi, T. , and Singh, A.K. (2017) A combined biochemical and computational studies of the rho‐class glutathione S‐transferase sll1545 of *Synechocystis* PCC 6803. Int J Biol Macromol 94(PtA): 378–385. 27760379 10.1016/j.ijbiomac.2016.10.040

[mbt213928-bib-0152] Parales, R.E. , Bruce, N.C. , Schmid, A. , and Wackett, L.P. (2002) Biodegradation, biotransformation, and biocatalysis (B3). Appl Environ Microbiol 68: 4699–4709. 12324310 10.1128/AEM.68.10.4699-4709.2002 PMC126401

[mbt213928-bib-0153] Park, H. , Vecitis, C.D. , Cheng, J. , Choi, J. , Mader, B.T. , and Hoffman, M.R. (2009) Reductive defluorination of aqueous perfluorinated alkyl surfactants: effects of ionic headgroup and chain length. J Phys L Chem A 113: 690–696. 10.1021/jp807116q 19123849

[mbt213928-bib-0154] Parsons, J.R. , Sáez, M. , Dolfing, J. , and De Voogt, P. (2008) Biodegradation of perfluorinated compounds. Rev Environ Contam Toxicol 196: 53–71. 19025092 10.1007/978-0-387-78444-1_2

[mbt213928-bib-0155] Pastore, A.J. , Teo, R.D. , Montoya, A. , Burg, M.J. , Twahir, U.T. , Bruner, S.D. , *et al*. (2021) Oxalate decarboxylase uses electron hole hopping for catalysis. J Biol Chem 297: 100857. 34097877 10.1016/j.jbc.2021.100857 PMC8254039

[mbt213928-bib-0156] Payne, K.A. , Quezada, C.P. , Fisher, K. , Dunstan, M.S. , Collins, F.A. , Sjuts, H. , *et al*. (2015) Reductive dehalogenase structure suggests a mechanism for B12‐dependent dehalogenation. Nature 517: 513–516. 25327251 10.1038/nature13901 PMC4968649

[mbt213928-bib-0157] Pearson, D.A. , Blanchette, M. , Baker, M.L. , and Guindon, C.A. (1989) Trialkylsilanes as scavengers for the trifluoroacetic acid deblocking of protecting groups in peptide synthesis. Tet Lett 30: 2739–2742.

[mbt213928-bib-0158] Peck, S.C. , Denger, K. , Burrichter, A. , Irwin, S.M. , Balskus, E.P. , and Schleheck, D. (2019) A glycyl radical enzyme enables hydrogen sulfide production by the human intestinal bacterium *Bilophila wadsworthia* . Proc Natl Acad Sci USA 116: 3171–3176. 30718429 10.1073/pnas.1815661116 PMC6386719

[mbt213928-bib-0159] Pedler, A.E. , Smith, R.C. , and Tatlow, J.C. (1972) The synthesis and dehydrofluorination of some polyfluoroalkanes. J Fluorine Chem 1: 337–345.

[mbt213928-bib-0160] Podder, A. , Sadmani, A.H.M.A. , Reinhart, D. , Chang, N.B. , and Goel, R. (2021) Per and poly‐fluoroalkyl substances (PFAS) as a contaminant of emerging concern in surface water: a transboundary review of their occurrences and toxicity effects. J Hazard Mater 419: 126361. 34157464 10.1016/j.jhazmat.2021.126361

[mbt213928-bib-0161] Poelarends, G.J. , and Whitman, C.P. (2004) Evolution of enzymatic activity in the tautomerase superfamily: mechanistic and structural studies of the 1,3‐dichloropropene catabolic enzymes. Bioorg Chem 32: 376–392. 15381403 10.1016/j.bioorg.2004.05.006

[mbt213928-bib-0162] Qin, J. , Chai, G. , Brewer, J.M. , Lovelace, L.L. , and Lebioda, L. (2006) Fluoride inhibition of enolase: crystal structure and thermodynamics. Biochemistry 45: 793–800. 16411755 10.1021/bi051558s PMC2566932

[mbt213928-bib-0163] Quack, B. , Peeken, I. , Petrick, G. , and Nachtigall, K. (2007) Oceanic distribution and sources of bromoform and dibromomethane in the Mauritanian upwelling. J Geophys Res 112: C10006.

[mbt213928-bib-0165] Renganathan, V. (1989) Possible involvement of toluene‐2,3‐dioxygenase in defluorination of 3‐fluoro‐substituted benzenes by toluene‐degrading *Pseudomonas* sp. strain T‐12. Appl Environ Microbiol 55: 330–334. 16347845 10.1128/aem.55.2.330-334.1989 PMC184110

[mbt213928-bib-0166] Rice, P.A. , Cooper, J. , Koh‐Fallet, S.E. , and Kabadi, S.V. (2021) Comparative analysis of the physicochemical, toxicokinetic, and toxicological properties of ether‐PFAS. Toxicol Appl Pharmacol 422: 115531. 33933458 10.1016/j.taap.2021.115531

[mbt213928-bib-0167] Richardson, P. (2021) Applications of fluorine to the construction of bioisosteric elements for the purposes of novel drug discovery. Expert Opin Drug Discov 10: 1–26. 10.1080/17460441.2021.1933427 34074189

[mbt213928-bib-0168] Richardson, R.E. (2013) Genomic insights into organohalide respiration. Curr Opin Biotechnol 24: 498–505. 23490446 10.1016/j.copbio.2013.02.014

[mbt213928-bib-0169] Rodrigues, A.V. , Tantillo, D.J. , Mukhopadhyay, A. , Keasling, J.D. , and Beller, H.R. (2020) Insight into the mechanism of phenylacetate decarboxylase (PhdB), a toluene‐producing glycyl radical enzyme. ChemBioChem 21: 663–671. 31512343 10.1002/cbic.201900560 PMC7079210

[mbt213928-bib-0170] Rutledge, H.L. , and Tezcan, F.A. (2020) Electron transfer in nitrogenase. Chem Rev 120: 5158–5193. 31999100 10.1021/acs.chemrev.9b00663 PMC7466952

[mbt213928-bib-0171] Sakakibara, S. , and Inukai, N. (1965) The trifluoroacetate method of peptide synthesis. I. The synthesis and use of trifluoroacetate reagents. Bull Chem Soc Japan 38: 1979–1984. 5856812 10.1246/bcsj.38.1979

[mbt213928-bib-0172] Sanger, F. (1945) The free amino groups of insulin. Biochem J 39: 507–515. 16747948 10.1042/bj0390507 PMC1258275

[mbt213928-bib-0173] Saunders, G.C. (1996) Defluorination of perfluoroalkanes and chlorofluorocarbons. Angewandte Chem Intll Ed Eng 35: 2615–2617.

[mbt213928-bib-0174] Scholtz, R. , Wackett, L.P. , Egli, C. , Cook, A.M. , and Leisinger, T. (1998) Dichloromethane dehalogenase with improved catalytic activity isolated from a fast‐growing dichloromethane‐utilizing bacterium. J Bacteriol 170: 5698–5704. 10.1128/jb.170.12.5698-5704.1988 PMC211671 3142855

[mbt213928-bib-0175] Schrauzer, G.N. , and Katz, R.N. (1978) Reductive dechlorination and degradation of mirex and kepone with Vitamin B12. Bioinorg Chem 9: 123–143. 81074 10.1016/s0006-3061(00)80285-9

[mbt213928-bib-0176] Schulz, E.C. , Mehrabi, P. , Müller‐Werkmeister, H.M. , Tellkamp, F. , Jha, A. , Stuart, W. , *et al*. (2018) The hit‐and‐return system enables efficient time‐resolved serial synchrotron crystallography. Nat Methods 15: 901–904. 30377366 10.1038/s41592-018-0180-2

[mbt213928-bib-0177] Schumacher, W. , and Holliger, C. (1996) The proton/electron ration of the menaquinone‐dependent electron transport from dihydrogen to tetrachloroethene in *Dehalobacter restrictus* . J Bacteriol 178: 2328–2333. 8636034 10.1128/jb.178.8.2328-2333.1996 PMC177941

[mbt213928-bib-0178] Seefeldt, L.C. , Yang, Z.Y. , Duval, S. , and Dean, D.R. (2013) Nitrogenase reduction of carbon‐containing compounds. Biochim Biophys Acta‐Bioenerget 1827: 1102–1111. 10.1016/j.bbabio.2013.04.003 PMC3714343 23597875

[mbt213928-bib-0179] Seffernick, J.L. , Johnson, G. , Sadowsky, M.J. , and Wackett, L.P. (2000) Substrate specificity of atrazine chlorohydrolase and atrazine‐catabolizing bacteria. Appl Environ Microbiol 66: 4247–4252. 11010866 10.1128/aem.66.10.4247-4252.2000 PMC92292

[mbt213928-bib-0180] Seffernick, J.L. , and Wackett, L.P. (2001) Rapid evolution of bacterial catabolic enzymes: a case study with atrazine chlorohydrolase. Biochemistry 40: 12747–12753. 11669610 10.1021/bi011293r

[mbt213928-bib-0181] Seong, H.J. , Kwon, S.W. , Seo, D.C. , Kim, J.H. , and Jang, Y.S. (2019) Enzymatic defluorination of fluorinated compounds. Appl Biol Chem 62: 1–8.

[mbt213928-bib-0182] Shaw, D.M. , Munoz, G. , Bottos, E.M. , Duy, S.V. , Sauvé, S. , Liu, J. , and Van Hamme, J.D. (2019) Degradation and defluorination of 6: 2 fluorotelomer sulfonamidoalkyl betaine and 6: 2 fluorotelomer sulfonate by *Gordonia* sp. strain NB4‐1Y under sulfur‐limiting conditions. Sci Total Environ 647: 690–698. 30092525 10.1016/j.scitotenv.2018.08.012

[mbt213928-bib-0183] Sheehan, D. , Meade, G. , Foley, V.M. , and Dowd, C.A. (2001) Structure, function and evolution of glutathione transferases: implications for classification of non‐mammalian members of an ancient enzyme superfamily. Biochemical J 360: 1–16. 10.1042/0264-6021:3600001 PMC1222196 11695986

[mbt213928-bib-0184] Sinclair, G.M. , Long, S.M. , and Jones, O.A.H. (2020) What are the effects of PFAS exposure at environmentally relevant concentrations? Chemosphere 258: 127340. 32563917 10.1016/j.chemosphere.2020.127340

[mbt213928-bib-0185] Singh, R.K. , Fernando, S. , Baygi, S.F. , Multari, N. , Thagard, S.M. , and Holsen, T.M. (2019) Breakdown products from perfluorinated alkyl substances (PFAS) degradation in a plasma‐based water treatment process. Environ Sci Technol 53: 2731–2738. 30768259 10.1021/acs.est.8b07031

[mbt213928-bib-0186] Slater, J.H. , Weightman, A.J. , and Hall, B.G. (1985) Dehalogenase genes of *Pseudomonas putida* PP3 on chromosomally located transposable elements. Mol Biol Evol 2: 557–567. 2835577 10.1093/oxfordjournals.molbev.a040366

[mbt213928-bib-0187] Smart, B.E. (2001) Fluorine substituent effects (on bioactivity). J Fluorine Chem 109: 3–11.

[mbt213928-bib-0188] Smidt, H. , Akkermans, A.D.L. , van der Oost, J. , and de Vos, W.M. (2000) Halorespiring bacteria–molecular characterization and detection. Enzyme Microb Techy 27: 812–820. 10.1016/s0141-0229(00)00316-1 11118591

[mbt213928-bib-0189] Smidt, H. , and de Vos, W.M. (2004) Anaerobic microbial dehalogenation. Annu Rev Microbiol 58: 43–73. 15487929 10.1146/annurev.micro.58.030603.123600

[mbt213928-bib-0190] Soiefer, A.I. , and Kostyniak, P.J. (1983) The enzymatic defluorination of fluoroacetate in mouse liver cytosol: the separation of defluorination activity from several glutathione S‐transferases of mouse liver. Arch Biochem Biophys 225: 928–935. 6625615 10.1016/0003-9861(83)90107-8

[mbt213928-bib-0191] Sonmez, B.B. , Zhang, Y. , Reuther, J.F. , Saleh, N.B. , Venkatesan, A.K. , and Apul, O.G. (2021) Thermal regeneration of spent granular activated carbon presents an opportunity to break the forever PFAS cycle. Environ Sci Technol 55: 5608–5619. 33881842 10.1021/acs.est.0c08224

[mbt213928-bib-0192] Stevens, J.C. (1991) Steroid derivatives as probes of adrenal cytochrome P450 structure and function. Ph.D. thesis, University of Arizona.

[mbt213928-bib-0193] Stockbridge, R.B. , Lim, H.H. , Otten, R. , Williams, C. , Shane, T. , Weinberg, Z. , and Miller, C. (2012) Fluoride resistance and transport by riboswitch‐controlled CLC antiporters. Proc Natl Acad Sci USA 109: 15289–15294. 22949689 10.1073/pnas.1210896109 PMC3458365

[mbt213928-bib-0194] Stotter, D.A. , Thomas, R.D. , and Wilson, M.T. (1977) Reductive dechlorination of DDT by haem proteins. Bioinorg Chem 7: 87–93. 192320 10.1016/s0006-3061(00)80130-1

[mbt213928-bib-0195] Studer, A. , Hadida, S. , Ferritto, R. , Kim, S.Y. , Jeger, P. , Wipf, P. , and Curran, D.P. (1997) Fluorous synthesis: a fluorous‐phase strategy for improving separation efficiency in organic synthesis. Science 275: 823–826. 9012347 10.1126/science.275.5301.823

[mbt213928-bib-0196] Sturr, M.G. , and Marquis, R.E. (1990) Inhibition of proton‐translocating ATPases of *Streptococcus mutans* and *Lactobacillus casei* by fluoride and aluminum. Arch Micobiol 155: 22–27. 10.1007/BF00291269 2150306

[mbt213928-bib-0197] Su, Y. , Rao, U. , Khor, C.M. , Jensen, M.G. , Teesch, L.M. , Wong, B.M. , *et al*. (2019) Potential‐driven electron transfer lowers the dissociation energy of the C‐F bond and facilitates reductive defluorination of perfluorooctane sulfonate (PFOS). ACS Appl Mater Interfaces 11: 33913–33922. 31436952 10.1021/acsami.9b10449

[mbt213928-bib-0198] Suflita, J.M. , Horowitz, A. , Shelton, D.R. , and Tiedje, J.M. (1982) Dehalogenation: a novel pathway for the anaerobic biodegradation of haloaromatic compounds. Science 218: 1115–1157. 17752871 10.1126/science.218.4577.1115

[mbt213928-bib-0199] Sun, H. , Keefer, C.E. , and Scott, D.O. (2011) Systematic and pairwise analysis of the effects of aromatic halogenation and trifluoromethyl substitution on human liver microsomal clearance. Drug Metab Lett 5: 232–242. 21916842 10.2174/187231211798472575

[mbt213928-bib-0200] Sun, Z. , Geng, D. , Zhang, C. , Chen, J. , Zhou, X. , Zhang, Y. , *et al*. (2021) Vitamin B12 (CoII) initiates the reductive defluorination of branched perfluorooctane sulfonate (br‐PFOS) in the presence of sulfide. Chem Eng J 423: 130149.

[mbt213928-bib-0201] Sunderland, E.M. , Hu, X.C. , Dassuncao, C. , Tokranov, A.K. , Wagner, C.C. , and Allen, J.G. (2019) A review if the pathways of human exposure to poly‐ and perfluoroalkyl substances (PFASs) and present understanding of the health effects. J Expo Sci Environ Epidemiol 29: 131. 30470793 10.1038/s41370-018-0094-1 PMC6380916

[mbt213928-bib-0202] Susarla, S. , Masunaga, S. , and Yonezawa, Y. (1997) Redox potential as a parameter to predict the reductive dechlorination pathway of chloroanilines in anaerobic environments. Micro Ecol 33: 252–256. 10.1007/s002489900028 9115189

[mbt213928-bib-0203] Sutton, S.V. , Bender, G.R. , and Marquis, R.E. (1987) Fluoride inhibition of proton‐translocating ATPases of oral bacteria. Infection Immun 55: 2597–2603. 10.1128/iai.55.11.2597-2603.1987 PMC259948 2889674

[mbt213928-bib-0204] Tahaikt, M. , El Habbani, R. , Haddou, A. , Achary, I. , Amor, Z. , Taky, M. , *et al*. (2007) Fluoride removal from groundwater by nanofiltration. Desalination 212: 46–53.

[mbt213928-bib-0205] Tang, S. , Wang, P.H. , Higgins, S.A. , Löffler, F.E. , and Edwards, E.A. (2016) Sister *Dehalobacter* genomes reveal specialization in organohalide respiration and recent strain differentiation likely driven by chlorinated substrates. Frontiers Microbiol 7: 100. 10.3389/fmicb.2016.00100 PMC4751268 26903979

[mbt213928-bib-0206] Thauer, R.K. , Zinkhan, D.M. , and Spormann, A.M. (1989) Biochemistry of acetate catabolism in anaerobic chemotrophic bacteria. Ann Rev Microbiol 43: 43–67. 2679359 10.1146/annurev.mi.43.100189.000355

[mbt213928-bib-0207] Thomas, A.W. , Lewington, J. , Hope, S. , Topping, A.W. , Weightman, A.J. , and Slater, J.H. (1992) Environmentally directed mutations in the dehalogenase system of *Pseudomonas putida* strain PP3. Arch Microbiol 158: 176–182. 1332636 10.1007/BF00290813

[mbt213928-bib-0208] Thomas, S.R. , McTamney, P.M. , Adler, J.M. , LaRonde‐LeBlanc, N. , and Rokita, S.E. (2009) Crystal structure of iodotyrosine deiodinase, a novel flavoprotein responsible for iodide salvage in thyroid glands. J Biol Chem 284: 19659–19667. 19436071 10.1074/jbc.M109.013458 PMC2740591

[mbt213928-bib-0209] Tiedt, O. , Mergelsberg, M. , Boll, K. , Müller, M. , Adrian, L. , Jehmlich, N. , *et al*. (2016) ATP‐dependent C‐F bond cleavage allows the complete degradation of 4‐fluoroaromatics without oxygen. Mbio 7: e00990‐16. 27507824 10.1128/mBio.00990-16 PMC4992971

[mbt213928-bib-0210] Tiedt, O. , Mergelsberg, M. , Eisenreich, W. , and Boll, M. (2017) Promiscuos defluorinating enoyl‐CoA hydratases/hydrolase allow for complete anaerobic degradation of 2‐fluorobenzoate. Front Microbiol 8: Article 2579. 10.3389/fmicb.2017.02579 PMC5742626 29312255

[mbt213928-bib-0211] Todd, M.J. , and Hausinger, R.P. (2000) Fluoride inhibition of *Klebsiella aerogenes* urease: mechanistic implications of a pseudo‐uncompetitive, slow‐binding inhibitor. Biochemistry 39: 5389–5396. 10820010 10.1021/bi992287m

[mbt213928-bib-0212] Turman, D.L. , Cheloff, A.Z. , Corrado, A.D. , Nathanson, J.T. , and Miller, C. (2018) Molecular interactions between a fluoride ion channel and synthetic protein blockers. Biochemistry 57: 1212–1218. 29393634 10.1021/acs.biochem.7b01272 PMC6215451

[mbt213928-bib-0213] Valverde, C. , Orozco, A. , Becerra, A. , Jeziorski, M.C. , Villalobos, P. , and Solís, J.C. (2004) Halometabolites and cellular dehalogenase systems: an evolutionary perspective. Int Rev Cytol 234: 143–199. 15066375 10.1016/S0074-7696(04)34004-0

[mbt213928-bib-0214] Visscher, P. , Culbertson, C. , and Oremland, R. (1994) Degradation of trifluoroacetate in oxic and anoxic sediments. Nature 369: 729–731.

[mbt213928-bib-0215] Vogel, T.M. , and McCarty, P.L. (1985) Biotransformation of tetrachloroethylene to trichloroethylene, dichloroethylene, vinyl chloride, and carbon dioxide under methanogenic conditions. Appl Environ Microbiol 49: 1080–1083. 3923927 10.1128/aem.49.5.1080-1083.1985 PMC238509

[mbt213928-bib-0216] Wackett, L.P. (2004) Evolution of enzymes for the metabolism of new chemical inputs in the environment. J Biol Chem 279: 41259–41262. 15187076 10.1074/jbc.R400014200

[mbt213928-bib-0217] Wackett, L.P. , and Hershberger, C.D. (2001) Biocatalysis and Biodegradation: Microbial Transformation of Organic Compounds. Washington, DC: ASM Press.

[mbt213928-bib-0218] Wackett, L.P. , and Robinson, S.L. (2020) The ever‐expanding limits of enzyme catalysis and biodegradation: polyaromatic, polychlorinated, polyfluorinated, and polymeric compounds. Biochem J 477: 2875–2891. 32797216 10.1042/BCJ20190720 PMC7428800

[mbt213928-bib-0219] Walker, M.C. , and Chang, M.C. (2014) Natural and engineered biosynthesis of fluorinated natural products. Chem Soc Rev 43: 6527. 24776946 10.1039/c4cs00027g

[mbt213928-bib-0220] Wang, S.L. , Rice, S.A. , Serra, M.T. , and Gross, B.R.I.O.N. (1986) Purification and identification of rat hepatic cytosolic enzymes responsible for defluorination of methoxyflurane and fluoroacetate. Drug Metab Disposition 14: 392–398. 2873984

[mbt213928-bib-0221] Wang, Y. , Chang, W. , Wang, L. , Zhang, Y. , Zhang, Y. , Wang, M. , *et al*. (2019) A review of sources, multimedia distribution and health risks of novel fluorinated alternatives. Ecotox Environ Safety 182: 109402. 10.1016/j.ecoenv.2019.109402 31280095

[mbt213928-bib-0222] Wang, Y. , and Liu, A. (2020) Carbon–fluorine bond cleavage mediated by metalloenzymes. Chem Soc Rev 49: 4906–4925. 32510080 10.1039/c9cs00740g PMC7375919

[mbt213928-bib-0223] Whitman, W.B. , Coleman, D.C. , and Wiebe, W.J. (1998) Prokaryotes: the unseen majority. Proc Natl Acad Sci USA 95: 6578–6583. 9618454 10.1073/pnas.95.12.6578 PMC33863

[mbt213928-bib-0224] Winchell, L.J. , Ross, J.J. , Wells, M.J.M. , Fonoll, X. , Norton, J.W. Jr , and Bell, K.Y. (2021) Per‐ and polyfluoroalkyl substances thermal destruction at water resource recovery facilities: a state of the science review. Water Environ Res 93: 826–843. 33190313 10.1002/wer.1483 PMC8375574

[mbt213928-bib-0225] Wischgoll, S. , Heintz, D. , Peters, F. , Erxleben, A. , Sarnighausen, E. , Reski, R. , *et al*. (2005) Gene clusters involved in anaerobic benzoate degradation of *Geobacter metallireducens* . Molecular Micro 58: 1238–1252. 10.1111/j.1365-2958.2005.04909.x 16313613

[mbt213928-bib-0226] Wiseman, A. (1970) Effect of inorganic fluoride on enzymes. In Pharmacology of Fluorides. Smith, F.A. (ed). Berlin, Heidelberg: Springer, pp. 48–97.

[mbt213928-bib-0227] Xie, Y. , Chen, G. , May, A.L. , Yan, J. , Brown, L.P. , Powers, J.B. , *et al*. (2020) *Pseudomonas* sp. strain 273 degrades fluorinated alkanes. Environ Sci Technol 54: 14994–15003. 33190477 10.1021/acs.est.0c04029

[mbt213928-bib-0228] Xu, Y. , Ge, Z. , Zhang, X. , Feng, H. , Ying, X. , Huang, B. , *et al*. (2019) Validation of effective roles of non‐electroactive microbes on recalcitrant contaminant degradation in bioelectrochemical systems. Environ Pollut 249: 794–800. 30951963 10.1016/j.envpol.2019.03.036

[mbt213928-bib-0229] Yang, Z.Y. , Dean, D.R. , and Seefeldt, L.C. (2011) Molybdenum nitrogenase catalyzes the reduction and coupling of CO to form hydrocarbons. J Biol Chem 286: 19417–19421. 21454640 10.1074/jbc.M111.229344 PMC3103320

[mbt213928-bib-0230] Yu, Y. , Zhang, K. , Li, Z. , Ren, C. , Chen, J. , Lin, Y.H. , *et al*. (2020) Microbial cleavage of C‐F bonds in two C6 per‐ and polyfluorinated compounds via reductive defluorination. Environ Sci Technol 54: 14393–14402. 33121241 10.1021/acs.est.0c04483

[mbt213928-bib-0231] Yue, Y. , Fan, J. , Xin, G. , Huang, Q. , Wang, J.B. , Li, Y. , *et al*. (2021) Comprehensive understanding of fluoroacetate dehalogenase‐catalyzed degradation of fluorocarboxylic acids: A QM/MM approach. Environ Sci Technol 55: 9817–9825. 34080849 10.1021/acs.est.0c08811

[mbt213928-bib-0232] Zhang, Q. , Dong, X. , Lu, J. , Song, J. , and Wang, Y. (2021) Chemoproteomic approach toward probing the interactomes of perfluoroalkyl substances. Anal Chem 93: 9634–9639. 34185510 10.1021/acs.analchem.1c01948 PMC8760635

[mbt213928-bib-0233] Zhao, Z. , Feng, Y. , Feng, H. , Ghulam, A. , Su, Y. , and Shen, D. (2014) Anaerobic biotransformation of fluoronitrobenzenes and microbial communities in methanogenic systems. J Environ Sci Health A Tox Hazard Subst Environ Eng 49: 1187–1197. 24844900 10.1080/10934529.2014.897537

[mbt213928-bib-0234] Zhu, J. , Xing, A. , Wu, Z. , Tao, J. , Ma, Y. , Wen, B. , *et al*. (2019) CsFEX, a fluoride export protein gene from *Camellia sinensis*, alleviates fluoride toxicity in transgenic *Escherichia coli* and *Arabidopsis thaliana* . J Agric Food Chem 67: 5997–6006. 31056906 10.1021/acs.jafc.9b00509

[mbt213928-bib-0235] Zimmerman, H.E. (2012) A mechanistic analysis of the Birch reduction. Acc Chem Res 45: 164–170. 21923089 10.1021/ar2000698

